# Identification of a Peptide-Pheromone that Enhances *Listeria monocytogenes* Escape from Host Cell Vacuoles

**DOI:** 10.1371/journal.ppat.1004707

**Published:** 2015-03-30

**Authors:** Bobbi Xayarath, Francis Alonzo, Nancy E. Freitag

**Affiliations:** Department of Microbiology and Immunology, University of Illinois at Chicago, Chicago, Illinois, United States of America; University of Michigan Medical School, UNITED STATES

## Abstract

*Listeria monocytogenes* is a Gram-positive facultative intracellular bacterial pathogen that invades mammalian cells and escapes from membrane-bound vacuoles to replicate within the host cell cytosol. Gene products required for intracellular bacterial growth and bacterial spread to adjacent cells are regulated by a transcriptional activator known as PrfA. PrfA becomes activated following *L*. *monocytogenes* entry into host cells, however the signal that stimulates PrfA activation has not yet been defined. Here we provide evidence for *L*. *monocytogenes* secretion of a small peptide pheromone, pPplA, which enhances the escape of *L*. *monocytogenes* from host cell vacuoles and may facilitate PrfA activation. The pPplA pheromone is generated via the proteolytic processing of the PplA lipoprotein secretion signal peptide. While the PplA lipoprotein is dispensable for pathogenesis, bacteria lacking the pPplA pheromone are significantly attenuated for virulence in mice and have a reduced efficiency of bacterial escape from the vacuoles of nonprofessional phagocytic cells. Mutational activation of PrfA restores virulence and eliminates the need for pPplA-dependent signaling. Experimental evidence suggests that the pPplA peptide may help signal to *L*. *monocytogenes* its presence within the confines of the host cell vacuole, stimulating the expression of gene products that contribute to vacuole escape and facilitating PrfA activation to promote bacterial growth within the cytosol.

## Author Summary

Experimental evidence has established that bacteria do not always exist as isolated single celled organisms, but instead communicate with each other through the secretion of small molecules that enable individual cells to coordinate complex traits and behaviors. Gram-positive bacteria rely on the secretion of small peptide pheromones to coordinate activities that include biofilm formation, exogenous DNA uptake via competence mechanisms, conjugal transfer of plasmid DNA, and expression of gene products that promote bacterial virulence. Here we provide evidence of a novel use of bacterial peptide pheromone signaling, that being the use of the pPplA peptide by *L*. *monocytogenes* to detect the confines of host cell vacuoles. Secretion and import of pPplA is required for efficient escape of *L*. *monocytogenes* from its initial membrane-bound host cell compartment, and bacteria lacking the peptide pheromone are severely attenuated for virulence in mice. The pPplA peptide pheromone is thus used by individual bacteria within a confined membrane bound space to coordinate the expression of gene products required by *L*. *monocytogenes* for intracellular growth and survival.

## Introduction

It has become increasingly apparent that bacteria do not simply exist as isolated, single celled organisms but instead have evolved a variety of communication systems that enable them to interact with other bacterial cells within a population. Bacterial cell-to-cell communication occurs through the secretion and sensing of small signal molecules that coordinate complex behaviors such as light emission, biofilm formation, DNA uptake and conjugation, bacteriocin synthesis, and virulence factor secretion [1–4]. For Gram positive bacteria, one important method of communication involves the synthesis of small peptide pheromones that are secreted by bacterial cells and trigger changes within the same cell or in receiver cells either via peptide import into the receiver or by stimulating membrane receptors that initiate signaling cascades within the receiver cell [1,3]. Four related but distinct peptide pheromone signaling systems have been described, and these include cyclical peptides exemplified by th e Agr signaling system, sensory systems of the RNPP family, signaling via peptides that contain Gly-Gly processing motifs, and peptides associated with the Rgg-like family of peptide-binding proteins that regulate gene expression [1].

Limited information has thus far been available regarding the use of peptide pheromone sensing to coordinate cellular functions of the Gram-positive environmental pathogen *Listeria monocytogenes* [5–9]. This bacterium survives as a saprophyte in soil but is capable of transitioning into life as an intracellular pathogen following ingestion by susceptible mammalian hosts [10–15]. Consumption of *L*. *monocytogenes* contaminated food products is the primary route of exposure in humans [16–20]; this normally leads to mild gastroenteritis in healthy individuals but can manifest into more serious invasive disease and even death in those who are immunocompromised, such as the elderly, individuals with HIV, chemotherapy and transplant patients, and in pregnant women, where infections can lead to stillbirths [12,15,21]. The ability for *L*. *monocytogenes* to cause disease in a mammalian host depends upon the expression of a number of gene products that enable *L*. *monocytogenes* to gain entry into host cells, escape from host cell vacuoles, replicate within the cytosol, and spread to infect adjacent cells [22–24]. Gene products contributing to host infection are up-regulated following bacterial entry into the host however the signal(s) that governs the induction of gene products associated with bacterial virulence remains unclear. It has been postulated that *L*. *monocytogenes* senses environmental cues that inform the bacterium as to its intracellular location [13].

One key factor that coordinates the transition of *L*. *monocytogenes* from a soil dweller into an intracellular pathogen is the central virulence regulatory protein, PrfA [10,13,25]. PrfA is a 27 kD transcriptional activator that regulates the expression of the majority of *L*. *monocytogenes* gene products associated with bacterial virulence [25–27]. It is a member of the cAMP receptor protein (Crp)-Fnr family of transcriptional regulators, many of which require the binding of a small-molecule cofactor for full activity [28–30]. PrfA regulates the expression of gene products that include those contributing to bacterial entry into non-professional phagocytic cells (InlA and InlB); those that mediate the perforation and disruption of host cell vacuoles [listeriolysin O (LLO) and the phospholipases PlcA and PlcB]; a protease that processes proPlcB to its active form (Mpl); a bacterial surface protein that directs host cell actin polymerization for bacterial movement (ActA); a hexose phosphate transporter that contributes to cytosolic growth (Hpt); and PrsA2, a post-translocation secretion chaperone that contributes to the folding and activity of *L*. *monocytogenes* secreted virulence factors [26,27]. PrfA activation and the induction of virulence gene expression occurs following bacterial entry into host cells, but the identity of the small molecule cofactor that is thought to trigger PrfA activation has not yet been determined. However, constitutively activated forms of the protein, known as PrfA* mutants, have been isolated and have been shown to have activity comparable to the fully activated wild type form that occurs within infected host cells [31–37].

We have examined changes in the profiles of secreted *L*. *monocytogenes* proteins following PrfA activation via prfA* mutations [38], and have recently identified a lipoprotein encoded by *lmo2637* whose secretion is increased in prfA* strains and that shares homology with a peptide pheromone-encoding lipoprotein whose associated pheromone regulates conjugal plasmid transfer in *Enterococcus faecalis* [39]. The Lmo2637 lipoprotein, or PplA (peptide pheromone-encoding lipoprotein A) is most homologous to the *E*. *faecalis* Cad lipoprotein; proteolytic processing of the Cad N-terminal secretion signal sequence gives rise to the cAD1 peptide that is imported into receiver cells and stimulates the expression of a number of gene products that result in bacterial aggregation and plasmid conjugal transfer [39–43]. Examination of the *L*. *monocytogenes* genome reveals a number of potential gene products that share homology with *E*. *faecalis* proteins that are required for peptide pheromone processing, import, and transcriptional regulation. Here we describe the functional analysis of the pPplA peptide pheromone in *L*. *monocytogenes* and its contributions to intracellular growth and bacterial virulence.

## Results

### Investigation of PplA as a peptide pheromone-encoding lipoprotein whose secretion is increased following PrfA activation

**Fig 1:**
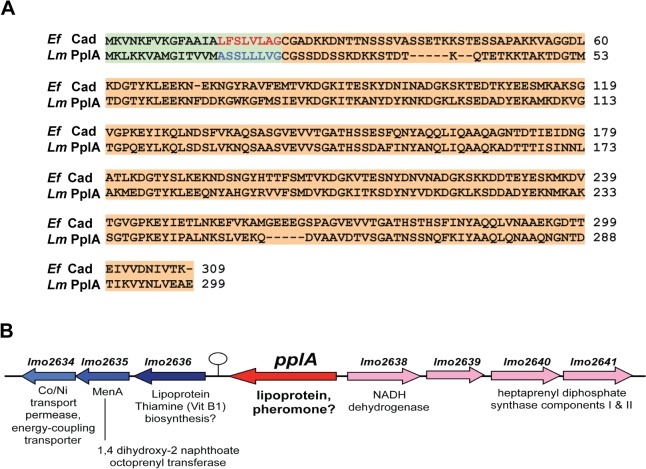
*L*. *monocytogenes* secretes a PrfA-induced lipoprotein that shares homology with *Enterococcus faecalis* Cad and its N-terminal encoded peptide-pheromone. **(A)** Protein alignment of *E*. *faecalis* Cad to *L*. *monocytogenes* Lmo2637 (PplA) using ClustalW2 software program (http://www.ebi.ac.uk/Tools/msa/clustalw2/). The signal sequence region encoding the cAD1 peptide-pheromone and the predicted pPlpA peptide are respectively highlighted in green and red for *Ef* (*E*. *faecalis*) or blue for *Lm* (*L*. *monocytogenes*), and the lipoprotein portion of the proteins is highlighted in tan. **(B)** Gene organization of the *pplA* coding region. The presence of a putative transcriptional terminator downstream of *pplA* is indicated by the circle with stem.

Mutational activation of the virulence regulatory protein PrfA (PrfA*) results in an increase in the abundance of a number of *L*. *monocytogenes* secreted proteins during growth in broth culture, including a gene product encoded by *lmo2637* [38], also known as *lmrg_02182* in strain 10403S. Lmo2637 is a putative peptide pheromone-encoding lipoprotein that shares significant homology over its entire sequence (50% identity, 66% similarity) with the *E*. *faecalis* Cad lipoprotein and its associated peptide pheromone cAD1 [39,43] (Fig. 1A). The functional role of the Cad lipoprotein is not known, however signal peptidase II-directed cleavage of the Cad N-terminal secretion signal sequence releases a 22 amino acid polypeptide that is further processed down to the eight amino acid peptide pheromone cAD1 [43]. cAD1 is imported via a peptide transport system into an *E*. *faecalis* receiver cell, where it binds to the TraA transcriptional repressor to relieve repression and stimulate expression of TraE1 [20,39,44]. TraE1 induces the expression of gene products that contribute to bacterial aggregation and conjugal transfer of plasmids into cAD1-peptide producer cells. In addition to Lmo2637, predicted gene products with homology to other *E*. *faecalis* components of the cAD1 pathway are also present, including one homologue of the Eep protease involved in the processing of cAD1 (Lmo1318, 50% identity and 66% similarity), two peptide transport systems (OppA and CtaP), and Lmo0833, a protein of which the first 60 amino acids are 34% identical and 49% similar to the N terminal DNA binding domain of the TraA repressor, while the C terminal region shares homology with Rgg peptide binding regulatory proteins (20% identity, 44% similarity to *Streptococcus pyogenes* Rggs). The *Listeria* genome encodes four additional Rgg-like proteins that all appear to have an N terminal DNA binding domain, however TraA shares the most homology with Lmo0833. In addition, the *Listeria* genome encodes a gene product that shares homology with the TraE1 activator, Lmo0618. Lmo0618 encodes a predicted protein of 380 amino acids, of which the first 115 are 24% identical and 45% similar to the entire 118 amino acid TraE1 protein, while the remaining C terminal portion of Lmo0618 contains a DUF872 domain, a conserved eukaryotic domain of unknown function.

Based on its shared homology with the Cad lipoprotein and cAD1 peptide pheromone, we have designated *lmo2637* as *pplA* for peptide pheromone-encoding lipoprotein A, encoding the mature lipoprotein PplA and the putative peptide pheromone pPplA. *pplA* is divergently transcribed relative to its neighbor *lmo2638*, which encodes a predicted NADH dehydrogenase (Fig. 1B). Within this region are genes that are predicted to encode subunits involved in the biosynthesis of menaquinone-7, a Vitamin K_2_ derivative and a thiamine (Vitamin B_1_) biosynthesis lipoprotein, a gene product similar to the *E*. *coli* MenA protein which is also involved in Vitamin K synthesis, a predicted membrane Co/Ni transport permease gene product similar to *B*. *subtilis* YbaF, and a large number of genes encoding both small and large ribosomal subunit proteins.

**Fig 2:**
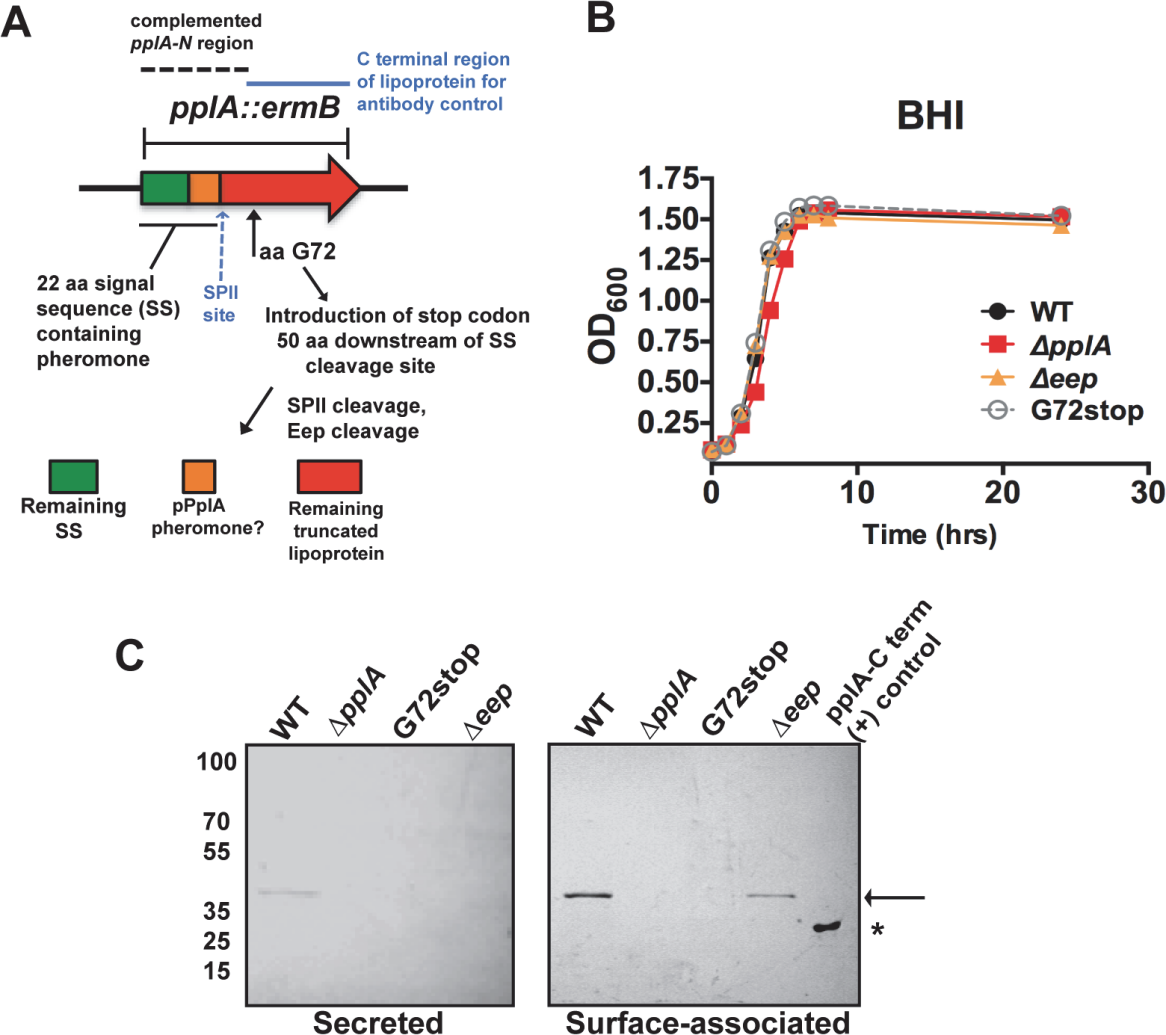
Construction of *L*. *monocytogenes* mutants that lack the PplA lipoprotein but retain peptide pheromone secretion. **(A)** Strategy for the construction of the in-frame *pplA* deletion and *pplA*-G72stop mutants that retain peptide pheromone secretion. The region used for complementation of the *pplA* deletion is indicated by the dashed lines. The C terminal lipoprotein region of PplA expressed and purified from *E*.*coli* that was used for affinity purification of the PplA antibody and also as the (+) control in western blots is indicated by the blue solid line. **(B)** Assessment of growth of the lipoprotein and pheromone mutants in BHI broth culture media. Overnight cultures of each strain grown shaking in BHI at 37°C were diluted 1:20 in fresh BHI media and the OD_600_ was measured at the indicated time points. **(C)** Western blot analysis of both surface-associated and secreted PlpA lipoprotein isolated from stationary phase cultures grown overnight shaking at 37°C. Samples were normalized to OD_600_. Secreted PplA was TCA extracted from the culture supernatant and surface-associated PplA was isolated by boiling in SDS-boiling buffer. Arrow indicates the position of full length PplA. ‘*’ indicates truncated and purified PplA lipoprotein used as positive control for antibody recognition. PplA lipoprotein is primarily detected in the surface-associated preparations versus the supernatant of wild-type *L*. *monocytogenes*. For panels (B) and (C), data is representative of at least three-independent experiments.

Peptide-pheromones secreted by *E*. *faecalis* coordinate conjugal plasmid transfer and contribute to bacterial virulence [1,39,45]. Given that the secretion of the PplA lipoprotein was increased following PrfA activation and that the majority of gene products associated with PrfA regulation contribute to bacterial virulence, we sought to investigate the function of the PplA lipoprotein and/or the pPplA peptide in *L*. *monocytogenes* physiology and mammalian infection. To differentiate between peptide pheromone secretion and production of the lipoprotein, two mutant constructs were generated: an in-frame deletion of the entire *pplA* coding sequence as well as a *pplA* mutant containing a stop codon introduced 50 amino acids downstream of the signal sequence cleavage site (G72_STOP_, Fig. 2A). To facilitate the transfer of the *pplA* deletion into different genetic backgrounds, the *ermB* gene encoding resistance to erythromycin was introduced in place of *pplA* coding sequences to enable the generation of *pplA* deletion mutants via phage transduction. The *pplA* mutant containing the engineered stop codon 50 amino acids downstream of the signal sequence cleavage site (named *pplA*-G72_STOP_) was designed so as to express only the peptide-pheromone and not the lipoprotein portion of PplA. A related mutation within *E*. *faecalis* Cad has been shown to truncate the lipoprotein while still enabling cAD1 peptide secretion [43]. In addition, an in-frame deletion of *eep* (*lmo1318*, encoding a predicted membrane-bound protease) was constructed to determine if Eep had any functional role associated with the proteolytic processing of the PplA N terminal signal sequence into mature pPplA peptide.

All mutants exhibited normal patterns of growth in BHI media (Fig. 2B) indicating that neither the lipoprotein, the lipoprotein and associated pheromone, or the Eep protease were required for growth in broth culture. Using an antibody directed against the C-terminal region of the PplA lipoprotein, western blot analysis confirmed the secretion of the lipoprotein in both surface-associated and secreted protein preparations derived from the wild-type strain 10403S (Fig. 2C). The PplA lipoprotein was not detected in protein preparations isolated from the Δ*pplA* mutant or the *pplA*-G72_STOP_ mutant (Fig. 2C). The PplA lipoprotein appeared more abundant in cell associated fractions versus bacterial supernatants (Fig. 2C, S1A Fig.). Both cell associated and secreted activities of Enterococcal lipoprotein-derived peptide-pheromones have been reported [46]. The presence of the PplA lipoprotein appeared modestly reduced in the Δ*eep* mutant, suggesting that Eep protease may directly or indirectly contribute to the stability of the mature PplA lipoprotein but is not absolutely required for PplA secretion.

The *E*. *faecalis* cAD1 peptide pheromone induces a mating response in plasmid-containing donor cells to stimulate conjugal transfer of plasmids to the recipient cells [40,47]. Production of a pheromone-induced aggregation substance (Asa1) on the cell surface of plasmid-containing cells enhances bacterial aggregation and conjugal transfer [48,49]. *E*. *faecalis* aggregation has been used as the basis for assays designed to detect the relative abundance of peptide-pheromone in culture supernatants from recipient cells or to test the activity of exogenously added synthetic peptides [40,47]. We and others have previously noted that prfA* cultures exhibit bacterial settling and/or aggregation when bacterial cultures are allowed to sit without shaking [37,50]. The physiological significance of this phenotype is not clear, although it has been recently reported that bacterial aggregation of prfA* strains is mediated by the surface protein ActA and contributes to persistence of intestinal colonization [50].

**Fig 3:**
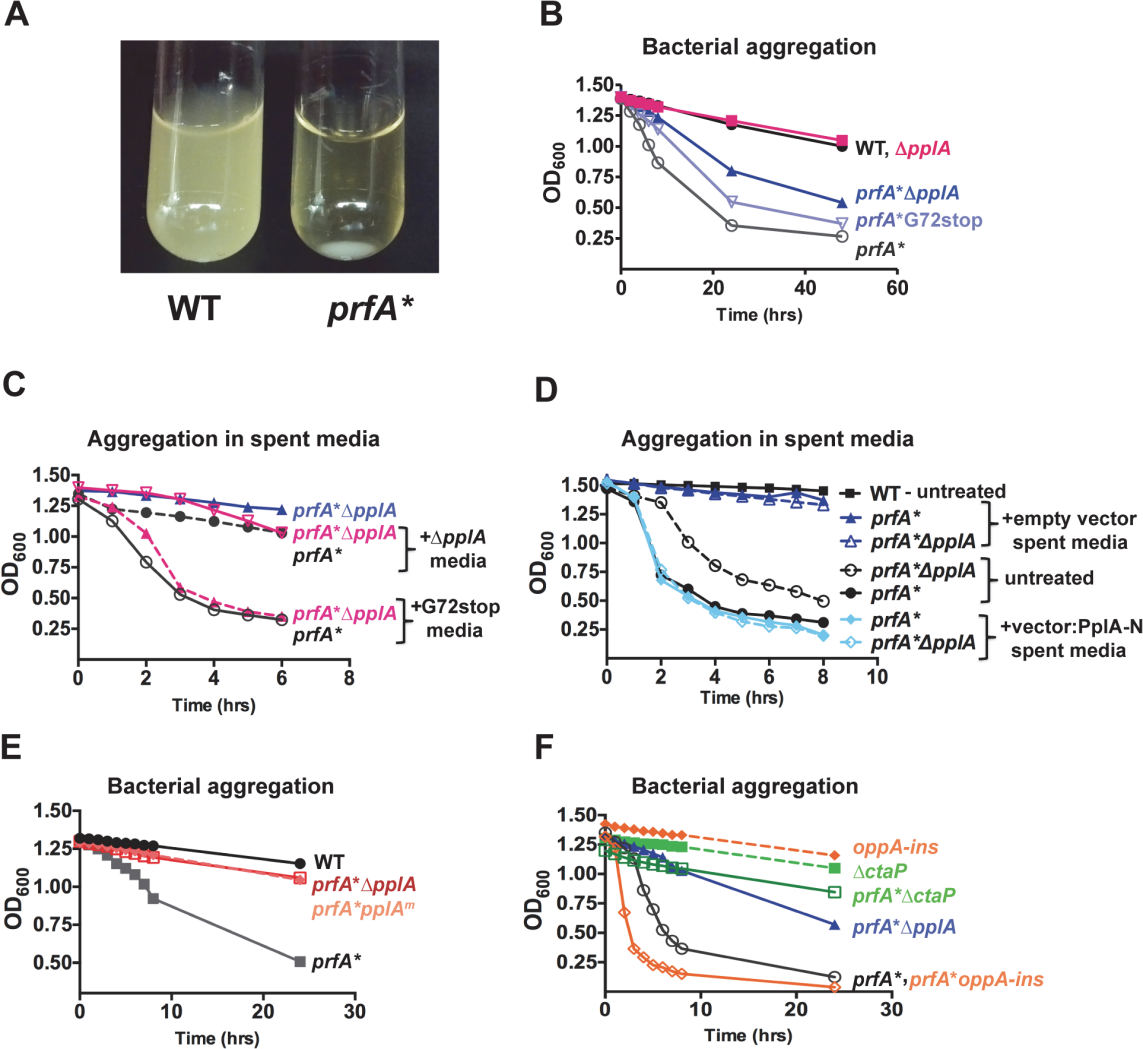
pPplA enhances bacterial aggregation in broth culture. **(A)** Image of bacterial aggregation observed between the wild-type *L*. *monocytogenes* 10403S strain versus a prfA* mutant when bacterial cultures grown in BHI are left statically overnight at room-temperature (RT). **(B)** Measurement of the rate of bacterial aggregation in BHI. The optical-density at 600nm was monitored at the indicated time points for 1 mL of an overnight culture initially grown in BHI with shaking at 37°C then left statically at RT, where bacterial aggregation is measured as the decrease in the optical-density of the culture supernatant as the bacterial aggregate out of solution. **(C)** Measurement of bacterial aggregation of the indicated mutant strains resuspended in 1 mL of spent media derived from overnight stationary phase cultures either containing the pPplA peptide (G72stop) or lacking it (Δ*pplA*). The ability of the pPplA containing media (G72stop) to restore bacterial aggregation indicates the presence of a secreted substance (potentially pPplA) that enhances bacterial aggregation in broth culture. **(D)** Measurement of bacterial aggregation as done in panel C, except strains were resuspended in 1 mL of BHI spent media derived from *E*. *coli* containing the complementation vector construct expressing the N-terminal 72 amino acids of PplA as described in Fig. 2A or the empty vector. The ability of spent media derived from an *E*.*coli* strain containing the first 72 amino acids of pPplA supports the secretion of a PplA N-terminus derived peptide that enhances bacterial aggregation. **(E)** Assessment of bacterial aggregation in a strain containing three amino acid substitutions within the predicted peptide sequence (referred to as prfA*pplA ^*m*^). Reduced aggregation of prfA*pplA ^*m*^ indicates the importance of these amino acids within the pPplA pheromone. **(F)** Bacterial aggregation of two oligopeptide transport mutants, a prfA* Δ*ctaP* compared to a prfA*-*oppA* insertion mutant. A prfA* Δ*ctaP* is impaired for bacterial aggregation, indicating a possible link between the pPplA peptide and import of the peptide through the CtaP transport system. For panels (B), (C) and (D), data is representative of at least three independent experiments.

To determine whether the pPplA pheromone contributed to bacterial aggregation of prfA* strains in broth culture, the Δ*pplA*::*erm* mutation was introduced into the prfA* L140F genetic background via phage transduction, and bacterial aggregation was determined by measuring aggregation-associated decreases in the optical density (Fig. 3B). Bacterial cultures were grown overnight at 37°C in BHI with shaking followed by static incubation at room temperature. prfA*L140F strains formed bacterial aggregates at the bottom of culture tubes after 24 hours of static incubation at room temperature, whereas the wild-type 10403S cultures remained in suspension (Fig. 3A). Interestingly, the prfA* Δ*pplA*::*erm* mutant exhibited reduced aggregation when compared to the parental prfA* strain (Fig. 3B). Although the ActA protein has been reported to contribute to the aggregation of prfA* cultures, the levels of ActA protein associated with the prfA* Δ*pplA*::*erm* mutant compared to the parental prfA*L140F strain were similar (S2A Fig.), suggesting that the presence of ActA was not sufficient for mediating aggregation of the prfA* Δ*pplA*::*erm* mutant. Introduction of the prfA* allele into strains containing the *pplA*-G72_STOP_ mutation resulted in aggregation patterns that were similar to the prfA* L140F strain (Fig. 3B). Spent culture media derived from a pheromone-producing strain (*pplA*-G72_STOP_), but not from Δ*pplA*::*erm*, could restore bacterial settling of the prfA* Δ*pplA*::*erm* mutant, consistent with the presence of a secreted substance derived from the N-terminal region of PplA enhancing bacterial aggregation in broth culture (Fig. 3C). Spent media treated with protease K did not restore bacterial aggregation to prfA* Δ*pplA*::*erm* cultures (S2B Fig.).

It has been previously shown that the expression of an *E*. *faecalis* N-terminal peptide pheromone coding region in *Escherichia coli* resulted in active pheromone secreted into the *E*. *coli* supernatant [51,52]. We therefore examined whether spent media derived from an *E*.*coli* strain containing a plasmid vector encoding the first 72 amino acids of the N-terminal region of PplA could restore aggregation to *L*. monocytogenes prfA* Δ*pplA* strains. Supernatants derived from *E*. *coli* containing the plasmid encoding *pplA*-G72_STOP_ restored aggregation to *L*. monocytogenes prfA* Δ*pplA* strains whereas supernatants derived from *E*. *coli* containing the plasmid vector alone did not (Fig. 3D). In addition, *L*. *monocytogenes* chromosomal mutants encoding the substitution of three residues within the predicted active peptide region of pPplA (ASSLLLVG to ASATLAVG, *pplA* ^m^ mutants) were observed to have reduced bacterial aggregation (Fig. 3E) while still maintaining secretion of the PplA lipoprotein (S1B Fig.). Confirmation of PplA secretion was important in this experiment given that the L20A substitution occurred within the conserved lipobox motif recognized by SPII [53] however sufficient conservation was retained to allow SPII cleavage and PplA secretion. Taken together, these results are consistent with the existence of a secreted pPplA peptide pheromone derived from the N-terminal secretion sequence of the PplA lipoprotein.

The cAD1 signaling peptide of *E*. *faecalis* is actively transported into recipient cells via the TraC oligopeptide transport system to stimulate the induction of gene products required for plasmid conjugation [39,54]. *L*. *monocytogenes* is predicted to encode five oligopeptide transport systems, two of which encode ABC transporter systems that are involved in the uptake of oligopeptides and three of which import di- and tri-peptides [55–58]. The oligopeptide OppA transport system *(lmo2196)* transports peptides that are 4–8 amino acids in length and is required for bacterial growth at low temperatures and for intracellular survival at early stages of infection in cultured macrophages and in mice [55]. The CtaP-associated system is an oligopeptide ABC transport system that also functions for high-affinity cysteine transport [58]. Oligopeptide-binding proteins associated with ABC transport systems have been reported to have either broad-specificity for peptide substrates or high sequence specificity [45,55,56,59]. We therefore examined whether the OppA or CtaP oligopeptide transport systems contributed to the transport of the pPplA pheromone-peptide by generating loss of function mutations within *oppA* and *ctaP* and examining patterns of bacterial aggregation in broth culture. prfA* *oppA* mutants exhibited patterns of bacterial aggregation that were similar to those of prfA*L140F strains, whereas the prfA* Δ*ctaP* mutant exhibited no bacterial aggregation (Fig. 3F). Production and secretion of the PplA lipoprotein was not impaired in the Δ*ctaP* mutant, although more lipoprotein was detected in the culture supernatant in comparison to cell associated proteins; this may be a reflection of changes surface hydrophobicity noted previously for this mutant (S1B Fig.) [58]. These results suggest that the CtaP ABC transporter system may be involved in the import of the pPplA pheromone into the bacterial cell to stimulate bacterial aggregation following PrfA activation.

### pPplA, but not the PplA lipoprotein, is required for bacterial virulence within mice

**Fig 4:**
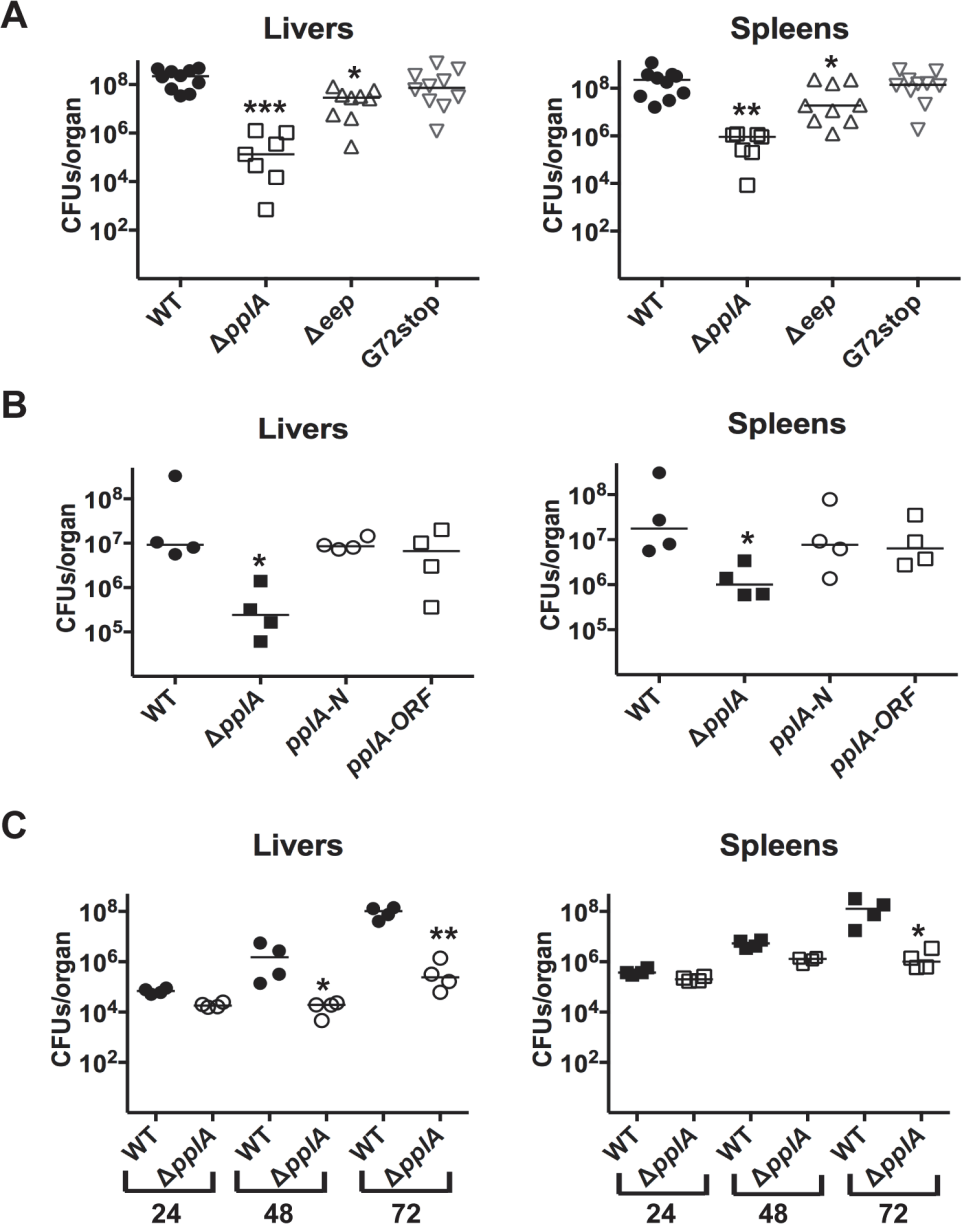
pPplA significantly contributes to bacterial virulence *in vivo*. **(A)** Swiss Webster female mice were intravenously inoculated with 2 x 10^4^ CFUs through the tail vein, and the livers and spleens were harvested 72 hours post-infection (p.i.) and homogenized to determine bacterial burdens. **(B)** Same as panel (A) except mice were also infected with the Δ*pplA* mutant complemented with either N-terminal of *pplA* (*pplA-N*) or the entire ORF (*pplA-ORF*). **(C)** Same as in panel (A), except the livers and spleens were harvested at 24, 48 and 72 hours p.i. to enable a comparison of bacterial burdens over time. Each datum point represents one mouse, and the solid horizontal line denotes the median for each data group. Data was obtained from two independent experiments. Asterisks indicated statistical significance of mutant strains in comparison to wild type *L*. *monocytogenes* using an unpaired two-tail student *t*-test (GraphPad Prism V.5.0A). *p≤0.05, **p≤0.005, and ***p≤0.0005.

The secretion of the PplA lipoprotein is increased following PrfA activation [38]. Given that a number of gene products directly or indirectly regulated by PrfA are known to contribute to bacterial virulence, we examined the phenotypes of mutant strains lacking the PplA lipoprotein, the pheromone and lipoprotein, and the Eep protease in a mouse infection model. Six to 8-week old female Swiss Webster mice were intravenously infected with 2 x10^4^ colony forming units of either wild-type 10403S, Δ*pplA*::*erm*, *pplA*-G72_STOP_, Δ*eep*, or the Δ*pplA*::*erm* mutant strain complemented with either the entire *pplA* open-reading frame or the N-terminal 72 amino acids encoded by *pplA* using the pIMK2 complementation vector, which integrates in single copy at a neutral tRNA^Arg^ site within the *L*. *monocytogenes* chromosome [35]. At three days post-infection the livers and spleens of infected mice were harvested to determine bacterial burdens. The Δ*pplA*::*erm* mutant was severely attenuated for virulence as mice infected with the mutant exhibited bacterial burdens that were two to three logs lower in comparison to mice infected with the wild-type strain (Fig. 4A). The Δ*eep* mutant was also significantly reduced for virulence in mice with bacterial burdens that were approximately one log lower in comparison to animals infected with wild-type *L*. *monocytogenes* (Fig. 4A). As Δ*eep* strains are less attenuated than Δ*pplA*::*erm* strains, this suggests that Eep may contribute to pPplA processing but is not absolutely required. In contrast, the *pplA*-G72_STOP_ mutant was fully virulent and exhibited no defects in colonization or replication within the liver or the spleen. Consistent with this result, the complemented Δ*pplA*::*erm* mutant containing either the entire *pplA* ORF or, most importantly, just the coding region for the N-terminal 72 amino acids fully complemented the virulence defect associated with the loss of *pplA* (Fig. 4B). These results strongly support a role for the N-terminal peptide pheromone of *pplA* (and not the PplA lipoprotein) in *L*. *monocytogenes* pathogenesis within the host.

Given that a virulence defect was detected for the *pplA* deletion mutant at three days post-infection, we sought to determine whether the mutant was defective for initial organ colonization, or whether the mutant was capable of colonizing host tissues but defective for replication. Female Swiss Webster mice were infected intravenously with wild-type *L*. *monocytogenes* or the Δ*pplA* mutant, and livers and spleens were harvested at 24, 48 and 72 hours post-infection. At 24 hours post-infection, the bacterial burdens detected within the livers and spleens were similar for mice infected with either wild-type 10403S or the Δ*pplA* mutant, indicating that the Δ*pplA* mutant demonstrated no significant defect in its ability to establish infections in target organs (Fig. 4C). However, differences in bacterial burdens became evident at 48 hours and increased by 72 hours post-infection, suggesting that the Δ*pplA* mutant was either defective for replication within tissues and/or cleared more rapidly by the host immune system.

### The pPplA pheromone enhances cytosolic bacteria replication within mammalian host cells

**Fig 5:**
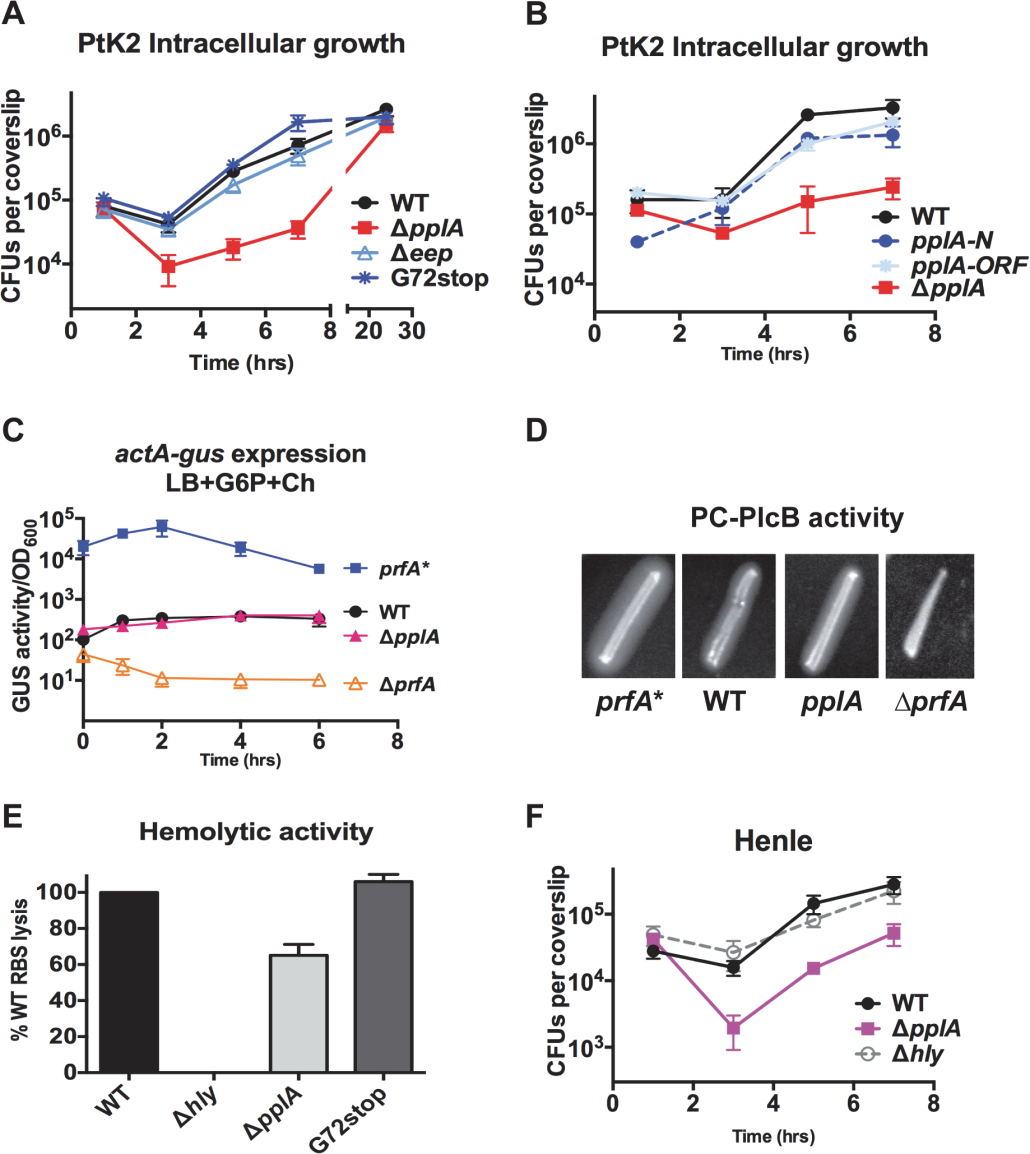
pPplA enhances *L*. *monocytogenes* vacuolar escape in host cells. **(A)** Intracellular growth of the indicated *L*. *monocytogenes* strains in PtK2 epithelial cells. PtK2 monolayers grown on glass coverslips (cs) were infected with bacteria at an MOI of 100:1. Gentamicin was added one hour p.i. to kill extracellular bacteria, cs were removed at indicated time points, host cells were lysed and the amounts of intracellular bacteria were enumerated. Loss of *pplA* delayed the initial stages of intracellular growth, suggestive of a vacuolar escape defect. **(B)** Intracellular growth in PtK2 cells (as described for panel A) of the Δ*pplA* mutant complemented with either the entire *pplA* open reading frame (*pplA-ORF*) or just the first 72 amino acids of the N terminal region (*pplA-N*). **(C)** Measurement of the level of *actA* and *plcB* expression in broth culture as assessed by monitoring strains containing *actA-gus-plcB* transcriptional reporter fusions. Bacterial strains were grown under *in vitro* inducing conditions for PrfA activity at 37°C with shaking, and GUS activity was measured from normalized samples collected at the indicated time points. Each data point represents the mean±SEM of GUS activity measured in duplicate from at least two independent experiments. **(D)** The production of PlcB-dependent phospholipase was assessed on egg yolk agar plates. Bacteria were streaked onto egg yolk plates and incubated overnight at 37°C. Zone of opacity surrounding bacterial streaks is indicative of PlcB activity. Loss of *pplA* did not impair PlcB-dependent phospholipase activity. Data shown is representative of three independent experiments. **(E)** Measurement of LLO-associated hemolytic activity as assessed by lysis of sheep red blood cells from serial dilutions of culture supernatants of bacterial strains grown shaking in LB for 5 hours at 37°C. Hemolytic activity was determined as the reciprocal of supernatant dilution at which 50% lysis was observed, the data is reported as the percentage of WT, with WT values set to 100%. **(F)** Intracellular growth assay in human Henle epithelial cell line, where vacuole escape is independent of LLO activity, done as described in panel (B) for PtK2 cells. For panels (A), (C) and (D), data shown represents the mean±SEM of three independent experiments done in triplicate.

The significant virulence attenuation observed for Δ*pplA* but not *pplA*-G72_STOP_ mutant strains was consistent with a role for pPplA in *L*. *monocytogenes* pathogenesis. We next examined the ability of mutants lacking pPplA to invade, replicate, and spread within host cell monolayers. No discernable defects were detected for mutant strains with respect to the invasion of PtK2 epithelial cells (Fig. 5A). However, the Δ*pplA* mutant exhibited a significant delay in intracellular replication following cell entry but by 24 hours post-infection reached intracellular numbers that were similar to those observed for wild type bacteria (Fig. 5A). In contrast to Δ*pplA*, the *pplA*-G72_STOP_ mutant and the Δ*pplA* strains complemented with either the full length *pplA* gene or just the N-terminal first 72 amino acids exhibited patterns of intracellular growth that were indistinguishable from cells infected with wild-type *L*. *monocytogenes* (Fig. 5AB). Similarly, the Δ*pplA* mutant exhibited a delay in cell-to-cell spread based on the formation of small zones of clearing or plaques in L2 fibroblast monolayers, whereas the *pplA*-G72_STOP_ mutant exhibited normal patterns of plaque formation (S3A and S3B Fig.). Interestingly, the Δ*eep* mutant displayed no significant defects for intracellular growth despite being attenuated for virulence (Fig. 5A). These data suggest that while the pPplA peptide pheromone is required for optimal bacterial replication within infected host cells, limited processing of the PplA N-terminal signal sequence-derived peptide may occur through the action of other proteases during intracellular infection.

### Loss of pPplA inhibits *L*. *monocytogenes* escape from host cell vacuoles in non-professional phagocytic cells

The delay observed in Δ*pplA* intracellular replication (versus *pplA*-G72_STOP_) suggested the possibility that strains lacking the pPplA peptide were defective in mediating escape from host cell vacuoles. Vacuole escape requires the secretion of the cholesterol-dependent pore-forming cytolysin listeriolysin O (LLO) and is assisted by two phospholipases, PlcA and PlcB [60–63]. Loss of *pplA* did not affect *plcB* expression based on an *actA-gus-plcB* transcriptional reporter gene fusion for which *β*-glucuronidase (GUS) activity serves as a read-out for *actA* and *plcB* expression (Fig. 5C), or secretion of PlcB as determined by examination of lecithinase activity on egg yolk agar plates (Fig. 5D). Modest reductions in secreted LLO activity were observed for the Δ*pplA* mutant as indicated by the reduced lysis of sheep red blood cells (about 70% of wild-type levels) (Fig. 5E). Modest reductions in secreted LLO activity have generally not been associated with significant vacuole escape defects as bacterial strains that exhibit approximately 10% LLO activity in comparison to wild type strains still exhibit normal patterns of vacuole escape and intracellular growth [64]. In addition, a delayed pattern of intracellular growth was also observed for the Δ*pplA* mutant in human Henle kidney epithelial cells for which vacuole lysis is not dependent on the activity of LLO but can be mediated by the activities of PlcB and PlcA (Fig. 5F) [65–68]. These results are consistent with a *L*. *monocytogenes* vacuole escape defect that is not restricted to variations in secreted LLO activity.

**Fig 6:**
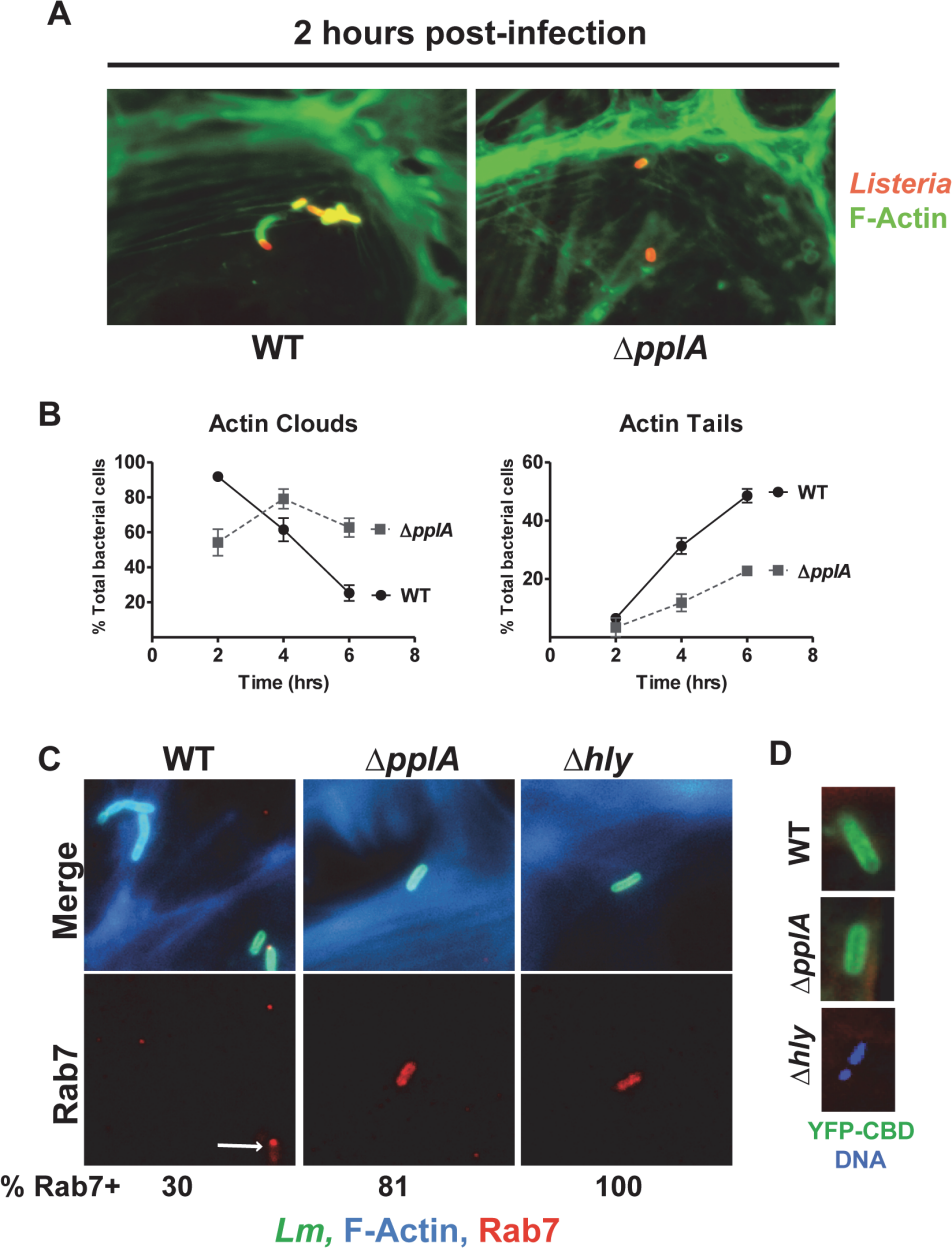
Loss of the pPplA pheromone delays escape from host cell vacuoles but does not impair vacuole perforation. **(A)** Host cell actin localization as a measure of cytosolic *L*. *monocytogenes* during infection of PtK2 epithelial cells. Monolayers of PtK2s were infected as described for Fig. 5, except that an MOI of 20:1 was used. At 2, 4, and 6 hours p.i., *Listeria* infected host cells were fixed and bacteria were stained using a *Listeria* specific polyclonal antibody, followed by a secondary goat anti-rabbit antibody conjugated to rhodamine (red stain). Host cell actin was stained using Alexa Fluor 488 phalloidin (green stain), which is a toxin that binds actin. Immunofluorescently labeled coverslips were then visualized on a Zeiss Axio Imager A2 microscope. Data shown is for 2 hours p.i. and is representative of 10 different fields from two independent experiments. **(B)** Quantification of actin clouds and actin tails formed by wild-type bacteria versus the Δ*pplA* mutant during intracellular growth in PtK2 cells. A total of 10 different fields, containing a total of 100 bacteria were assessed for clouds and tails. A Δ*pplA* mutant was delayed in recruitment of host-cell actin, consistent with a vacuole escape defect. **(C)** Co-localization studies of bacterial cells with host cell Rab7, a small GTPase associated with the late endosome. Ptk2 cells were infected and processed for microscopy as described above except that coverslips were removed at 1.5 hours post-infection and host cell F-actin was stained with phalloidin conjugated to Alexa-350 (blue), the secondary antibody used to stain *Listeria* cells was conjugated to Alexa-488 (green), and host cell Rab7 was stained with goat anti-Rab7 followed by a secondary donkey anti-goat antibody conjugated to Texas Red (red). Both the Δ*hly* and Δ*pplA* mutants stained robustly with Rab7 and the majority of Δ*pplA* bacteria counted co-localized with Rab7, whereas only a small number of wild-type *Listeria* were positive for Rab7. A population of wild type bacteria also formed actin clouds at this early time. These results suggest the loss of the pPplA peptide results in bacterial mutants that are retained within vacuoles and which are delayed for entry into the cytosol. A minimum of 10 different fields containing a total of at least 100 bacteria in two independent experiments were counted for each strain. **(D)** The contribution of the pPplA peptide to vacuole membrane perforation was assessed during intracellular growth in PtK2 cells. PtK2 cells were transfected with a mammalian expression vector containing a yellow fluorescent protein (YFP) fused to the cell wall binding domain of a phage endolysin Ply118 (CBD) that binds with high affinity to the *Listeria* cell surface. YFP-CBD is stably expressed in the host cytosol and nucleus, and once intracellular *L*. *monocytogenes* perforates the vacuole membrane, YFP-CBD enters the vacuole and binds bacteria prior to escape into the cytosol. Transfected PtK2 cells were infected with bacteria as described for panel A, except coverslips were removed at 15 minutes post-infection and host cell-F-actin was stained with phalloidin conjugated to Texas Red (red), DNA with DAPI (blue), and bacteria were green if bound with YFP-CBD. Data shown is representative of three independent experiments. YFP-CBD binding of wild-type and the Δ*pplA* mutant but not Δ*hly* bacteria indicates that vacuole perforation was not impaired by the loss of the pPplA peptide.

*L*. *monocytogenes* mutant strains that exhibit vacuole escape defects are delayed in gaining access to the host cytosol, delayed in initiating bacterial cytosolic replication, and delayed in the acquisition and polymerization of host cell actin, a process that is dependent on the expression of the bacterial surface protein ActA [31,69–71]. Defects in vacuole escape can be monitored based on the timing of bacterial accumulation of host cell actin filaments within infected cells using fluorescent indicators for F-actin. Following the infection of PtK2 epithelial cells, Δ*pplA* exhibited a significant delay in vacuolar escape based both on its reduced rate of host actin accumulation as well as its failure to initiate bacterial replication (replication does not occur within host vacuoles but is restricted to the cytosol [72,73]) (Fig. 6A). In contrast, at 2 hours post-infection cells infected with the wild-type strain co-localized with host cell actin as indicated by the formation of actin clouds surrounding individual bacteria. A small percentage of wild-type bacteria formed short actin tails indicative of subsequent steps of polar ActA localization and bacterial movement. In contrast, the Δ*pplA* mutant either failed to co-localize with host cell actin or exhibited reduced numbers of bacteria associated with actin clouds and minimal actin tail formation. Quantification of the total numbers of actin clouds formed versus tails over time conclusively demonstrated the delay of the Δ*pplA* mutant in accumulating host cell actin and initiating movement (Fig. 6B).

A defect in vacuolar escape for mutants lacking pheromone was further verified by observing that the Δ*pplA* mutants co-localized with Rab7 (a small GTPase associated with late endosomal vacuoles) at an early time point post-infection in infected PtK2 cells. Examination of infected cells at 1.5 hours post-infection revealed co-localization of Rab7 with the Δ*pplA* mutant as well as with a *L*. *monocytogenes* Δ*hly* mutant that lacks LLO and remains trapped within host vacuoles (Fig. 6C). 100% of the Δ*hly* mutants co-localized with Rab7, whereas approximately 81% of Δ*pplA* mutants were positive for Rab7 and only 30% of wild type bacteria co-localized with Rab7 at 1.5 hours post-infection. Furthermore, wild type bacteria that did stain positive for Rab7 exhibited reduced or punctate staining patterns, suggesting that the bacteria were losing the endosomal marker (Fig. 6C). These data support a role for the pPplA peptide in mediating efficient vacuole escape.

### Loss of the pPplA peptide does not impair vacuole membrane perforation

While the loss of the pPplA peptide delayed host cell vacuole escape, it appeared that significant levels of proteins associated with vacuole lysis, LLO and phospholipase, were secreted in broth culture (Fig. 5C-F). LLO in particular is associated with rapid perforation of host cell vacuoles [74], thus we sought to determine whether membrane perforation itself was impaired by loss of pPplA, or alternatively if membrane perforation occurred but bacterial escape was inhibited. Based an approach developed by Henry *et al* [74], PtK2 cells were transfected with a mammalian expression vector encoding a fusion protein of yellow fluorescent protein (YFP) fused the cell wall binding domain (CBD) of the phage endolysin Ply118 which binds to the *L*. *monocytogenes* cell wall with high affinity [74]. YFP-CBD protein is stably expressed in the host cell cytosol and nucleus in transfected cells; when vacuole perforation occurs as a result of *L*. *monocytogenes* host cell infection, YFP-CBD from the cytosol enters the vacuole and binds to the bacterium. Binding of YFP-CBD to intracellular *L*. *monocytogenes* is thus an early readout of vacuolar membrane perforation and can be detected prior to bacterial entry into the cytosol [74].

Transfected PtK2 cells expressing YFP-CBD were grown on glass coverslips and infected with either wild type *L*. *monocytogenes*, the Δ*pplA* or the Δ*hly* mutant. Cells were examined using fluorescence-based microscopy at 15 and 30 minutes post-infection. In macrophages, LLO-dependent vacuole membrane perforation can be detected within 5 minutes following bacterial internalization, whereas complete vacuole escape generally occurs within about 30–45 minutes [74,75]. Both wild type *L*. *monocytogenes* and the Δ*pplA* mutant were observed to bind YFP-CBD by 15 minutes post-infection, indicating that Δ*pplA* mutant was capable of perforating the vacuole membrane (Fig. 6D). Mutants trapped within host vacuoles due to lack of LLO secretion (Δ*hly*) did not stain with YFP-CBD (Fig. 6D). These results indicate that the pPplA peptide contributes to *L*. *monocytogenes* escape from host cell vacuoles at a step that is subsequent to initial vacuole membrane perforation.

### pPplA is dispensable for intracellular growth inside professional phagocytic cells

**Fig 7:**
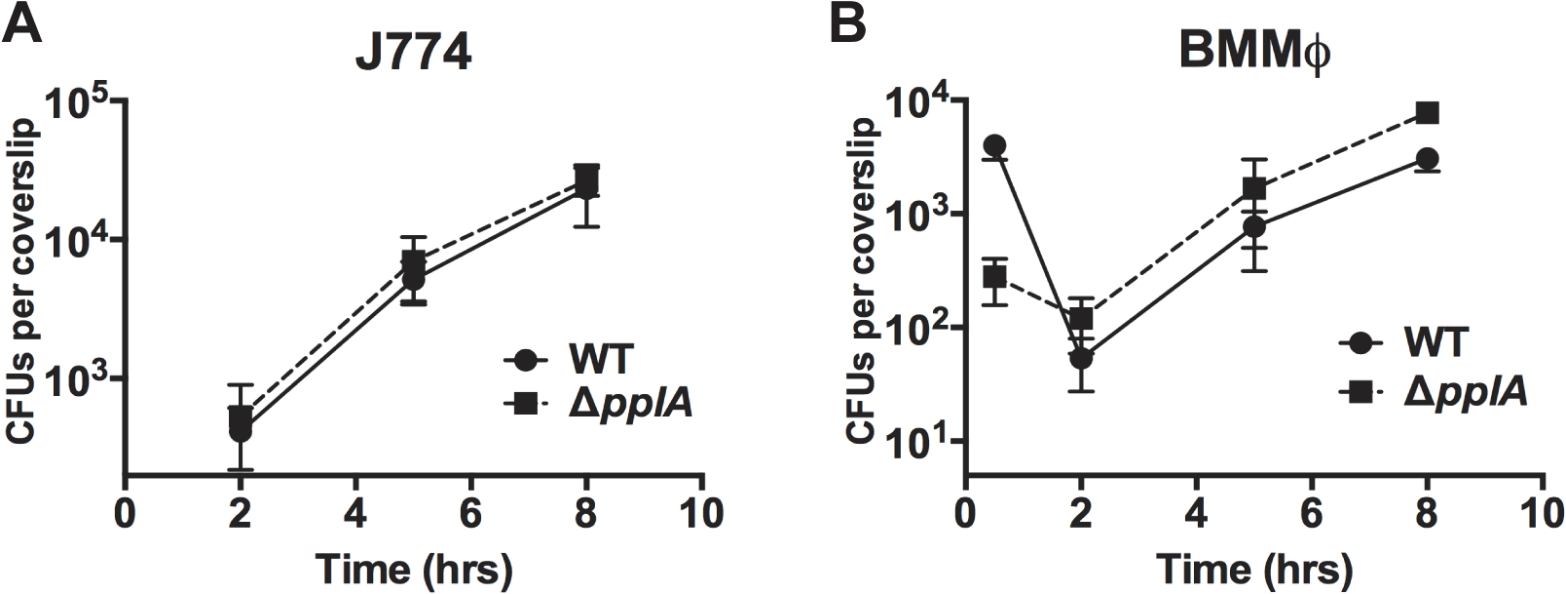
Loss of *pplA* pheromone does not delay intracellular growth or vacuolar escape in professional phagocytic cells. Intracellular growth of the wild-type strain compared to the Δ*pplA* mutant in (A) J774 macrophage-like cells and (B) murine bone-marrow derived macrophages (BMMØ) using an MOI of 01:1. Loss of *pplA* did not impair intracellular growth inside professional phagocytic cells. Data shown is representative of three independent experiments.

Resident macrophage populations are often the first cells to encounter *Listeria* circulating in the blood [76]. Surprisingly, when bacterial growth was examined following the infection of J774 murine macrophage-like cells, the growth pattern of the Δ*pplA* mutant resembled that of wild type bacteria, with no discernable defect in phagosomal escape (Fig. 7A). Similarly, the infection of primary bone-marrow derived macrophages revealed no phenotypic difference between mutant and wild type bacterial strains, even following macrophage activation with IFNγ(Fig. 7B and S4 Fig.). As mentioned above, resident macrophages are usually the cells associated with the initial contact of *L*. *monocytogenes* circulating in the bloodstream. Therefore, these results support the *in vivo* time course data that indicated that the Δ*pplA* mutant was not impaired in its ability to initially colonize target organs. The pPplA peptide thus enhances bacterial escape from the vacuoles of non-professional phagocytic cells but appears dispensable for growth within professional phagocytic cells.

### Constitutive activation of the PrfA regulator restores virulence to strains lacking the pPplA peptide pheromone

**Fig 8:**
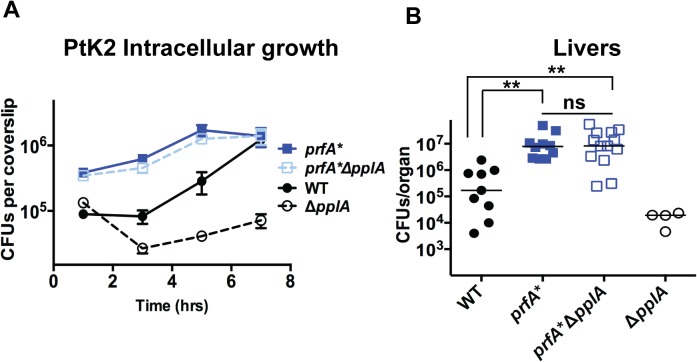
Constitutive activation of prfA* rescues virulence defects associated with loss of pPplA pheromone. **(A)** Intracellular growth of the prfA* mutant, the wild-type strain and a prfA*Δ*pplA* mutant in PtK2 epithelial cells. Gentamicin was added one hour p.i. to kill extracellular bacteria, coverslips were removed at the indicated time points, host cells were lysed and intracellular bacteria were enumerated. **(B)** Swiss Webster female mice were intravenously inoculated with 2 x 10^4^ CFUs through the tail vein, and the livers and spleens were harvested 48 hours post-infection (p.i.) and homogenized to determine bacterial burdens. The Δ*pplA* single mutant data is from data presented in Figs. 4 and 5 and is meant to represent a point of reference. The addition of a prfA* mutation to the Δ*pplA* mutant is able to completely restore any virulence defects associated with loss of *pplA*, suggesting a link between the pPplA pheromone function and PrfA activation.

The transcriptional activator PrfA regulates the expression of a number of gene products required for *L*. monocytogenes pathogenesis, including the process of vacuole escape [25–27]. PrfA is a member of the cAMP receptor protein (Crp)-Fnr family of transcriptional regulators that require the binding of small molecule cofactors to become fully activated for transcription [28,30,32]. The putative PrfA cofactor is unknown, but it has been determined that PrfA activation occurs following the entry of *L*. *monocytogenes* into host cells. Given the apparent vacuole escape defect of strains lacking the pPplA secreted peptide, we investigated whether mutational activation of PrfA could compensate for the intracellular growth defect resulting from the loss of *pplA* and/or the virulence defect observed in mice. The introduction of the mutationally activated prfA*L140F allele completely rescued the growth defect observed for the *pplA* deletion mutation within infected host cells and also fully restored virulence in mice to the levels of prfA* strains, which are hypervirulent in comparison to wild type *L*. *monocytogenes* (Fig. 8AB). Mutational activation of *prfA* therefore fully compensated for the loss of the pPplA secreted peptide, suggesting a possible linkage between pPplA function and PrfA activation.

### *L*. *monocytogenes* mutants that lack pPplA exhibit altered profiles of surface and secreted proteins

Peptide pheromone secretion and signaling pathways have been associated with the induction of selected gene products in a variety of Gram positive bacteria. For example, peptide pheromone signaling has been shown to regulate competence and sporulation in *Bacillus subtilis* [77,78], conjugation [39,40,45,47,79] and virulence factors/biofilm formation [80–82] in *Enterococcus*, competence and fratricide in *Streptococcus pneumoniae* [83,84], and virulence factor secretion in *Staphylococcus aureus* [4,85,86]. We therefore investigated whether the pPplA peptide pheromone influenced patterns of *L*. *monocytogenes* secreted proteins by examining polypeptide profiles following two-dimensional gel electrophoresis with subsequent peptide identification using mass spectroscopy. Proteins were isolated from strains grown in BHI to stationary phase as stationary phase-derived supernatants were observed to contain more peptide activity to promote bacterial aggregation, and also based on the abundance of the PplA lipoprotein as monitored by Western blot analyses using antibody directed against PplA. The comparison of secreted protein profiles for wild type, the *pplA*-G72_STOP_ mutant, and Δ*pplA* facilitated the identification of changes in protein profiles specific to the loss of the peptide pheromone versus the PplA lipoprotein.

**Fig 9:**
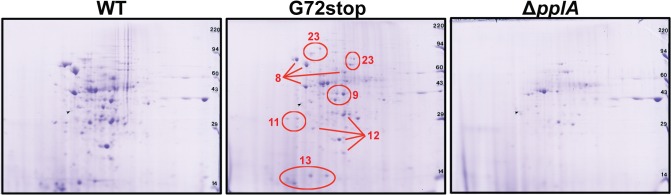
Loss of pPplA affects bacterial protein secretion. Two dimensional gel electrophoresis of secreted protein preparations isolated from overnight stationary phase cultures grown in BHI. Secreted proteins were TCA precipitated from bacterial culture supernatants and processed for 2-D gel analysis by Kendrick Labs (Madison, WI). The wild-type and the *pplA*-G72stop mutant protein profiles displayed similar patterns, whereas loss of *pplA* resulted in the loss of a number of secreted proteins.

Dramatic differences in the profiles of secreted proteins were observed for the *pplA* in-frame deletion mutant in comparison to wild-type 10403S and *pplA*-G72_STOP_ strains (Fig. 9). A number of proteins secreted by 10403S and *pplA*-G72_STOP_ were absent in the supernatants derived from the Δ*pplA* mutant, while the secreted protein profiles of wild-type 10403S and *pplA*-G72_STOP_ were similar. Complementation of the Δ*pplA* mutant with the first 72 amino acids of PplA restored secreted protein profiles to those resembling wild type (S1A Fig.). In contrast, the protein profiles of supernatants derived from prfA* L140F cultures appeared similar to those of prfA*L140F Δ*pplA* and prfA*L140Fx*pplA*-G72_STOP_ (S5A Fig.), a finding consistent with the ability of the constitutively activated prfA* allele to compensate for loss of the secreted pPplA peptide within cells and *in vivo*.

As loss of the *pplA*-encoded pheromone and not the encoded lipoprotein significantly diminished *L*. *monocytogenes* pathogenesis, we chose to focus on most significant differences in secreted protein profiles between the *pplA*-G72_STOP_ mutant versus the *pplA* in-frame deletion mutant (summarized in Table 1). Secreted proteins absent from the culture supernatants of strains lacking pPplA were associated with a range of functional groups that included substrate-binding lipoproteins involved in metal and oligopeptide ABC transport (OppA), protein folding, adaptation to atypical conditions, detoxification, membrane bioenergetics, and also a number of metabolic and amino acid biosynthetic proteins. The majority of these proteins do not appear to be associated with SecA1-dependent protein secretion as they lack the presence of a classical secretion signal peptide, however a significant number have been associated with SecA2-dependent secretion [87–89]. We confirmed that the presence of proteins with cytosolic function was not the result of increased bacterial cell lysis as cell viability based on bacterial CFU was identical between cultures, and there was no indication of compromised membrane integrity as visualized by cell impermeant dyes (S5B Fig.). While it is not yet clear as to how the pPplA-dependent secretion of these proteins enhances *L*. *monocytogenes* vacuolar escape, it is evident that pPplA-dependent signaling leads to significant changes in the repertoire of *L*. *monocytogenes* secreted and surface-associated proteins.

**Table 1:** Secreted protein spots present in both wild-type 10403S and the *pplA*-G72_STOP_ mutant but absent in the in-frame *pplA* deletion mutant.

Protein ID^*a*^	Description	Secretion system^*b*^	Peptide Matches^*c*^	% Sequence coverage	MW (kDa)
**Group 8**					
Lmo2068	Chaperonin GroEL—protein folding during stress conditions	SecA2	22	62.20	57
Lmo1473	Chaperone DnaK—protein folding, hyperosmotic stress	SecA2	17	56.10	66
Lmo2455	Enolase—phosphopyruvate hydratase	SecA2	21	64.00	46
Lmo1055	Dihydrolipoamide Dehydrogenase (DH)—part of pyruvate DH complex		9	28.90	49
Lmo0355	Fumerate reductase flavoprotein subunit	SecA	7	24.70	54
Lmo1620	Dipeptidase PepV—degrades hydrophobic peptides		11	35.10	52
Lmo0135	CtaP—lipoprotein, substrate-binding protein	SecA	13	46.40	58
Lmo0644	Hypothetical lipoprotein	SecA	4	30	51
**Group 9**					
Lmo2459	Glyceraldehyde-3-phosphate dehydrogenase	SecA2	16	74.40	36
Lmo0539	Tagatose 1,6-diphosphate aldolase		12	52.70	38
Lmo2659	Dihydroacetone kinase		8	52.30	35
Lmo0554	Oxidoreductase		11	45.00	43
Lmo2416	Lipoprotein	SecA	4	21.20	40
**Group 11**					
Lmo2415	FeS Assembly ATPase SufC		12	55.20	29
Lmo9211	Ctc—general stress protein	SecA2	9	62.80	23
Lmo1474	Heat shock protein GrpE		5	64.40	22
Lmo1571	6-phosphofructokinase	SecA2	5	30.40	32
Lmo0223	Cysteine Synthase		5	26.00	32
Lmo2556	Fructose-1,6-bisphophate aldolase		7	48.90	30
Lmo2459	Glyceraldehyde 3-phosphate dehydrogenase	SecA2	8	33.90	36
**Group 12**					
Lmo1439	Superoxide dismutase	SecA2	14	86.10	23
Lmo0698	Flagellin	SecA2	12	61.00	30
Lmo1583	Thiol peroxidase		5	57.60	18
Lmo2511	Similar to sigma 54		6	49.20	22
Lmo0191	Similar to phospho-beta-glucosidase		5	32.20	27
Lmo2256	Protease I		9	71.10	19
**Group 13**					
Lmo0943	Iron-binding ferritin, oxidative damage protection		8	64.70	18
Lmo1233	Thioredoxin	SecA2	6	61.20	12
Lmo1439	Superoxide dismutase	SecA2	9	60.90	23
Lmo1658	50S ribosomal protein L7/L12		5	63.30	12
**Group 23**					
Lmo2654	Elongation factor G	SecA2	18	40.30	77
Lmo1570	Pyruvate kinase	SecA2	16	50.40	63
Lmo1293	Glycerol 3-phosphate dehydrogenase	SecA2	16	37.60	63
Lmo1305	Transkelotase		14	31.90	72
Lmo2196	OppA—oligopeptide substrate-binding protein	SecA	12	32.60	63

^*a*^EGD-e designations.

^*b*^SecA2 secreted proteins identified in Lenz *et al* [88] and in Renier *et*. *al* [89] or possibly secreted by the SecA2 system as these proteins have been identified as non-classically secreted proteins in Bendtsen *et al* [87] or by using SecretomeP 2.0 Server program (http://www.cbs.dtu.dk/services/SecretomeP/); SecA secreted proteins identified in Port and Freitag [38] and/or Baumgartner *et*. *al* [103], or predicted by SignalP 4.1 Server program (http://www.cbs.dtu.dk/services/SignalP/).

^*c*^Unique peptide matches.

## Discussion

Bacterial peptide pheromones have been described as signaling molecules that coordinate bacterial communication and complex multicellular processes that include biofilm formation, DNA transfer via conjugation, as well as virulence factor secretion [1,2,4]. Here we present evidence for a novel signaling role for a bacterial peptide pheromone, namely facilitating the escape of an individual bacterial cell from the confines of a host cell vacuole. Experimental evidence suggests that the pPplA peptide pheromone is released by *L*. *monocytogenes* following its cleavage and processing from the secretion signal sequence of the PplA lipoprotein; production of the pPplA peptide enhances *L*. *monocytogenes* escape from host cell vacuoles and is required for full bacterial virulence. Our data suggests that pPplA may serve to signal to *L*. *monocytogenes* that the bacterium is within the confines of a membrane-bound vacuole, such that the secretion of pPplA leads to its rapid uptake to initiate a signaling cascade that ultimately enhances vacuolar membrane dissolution and bacterial escape into the cytosol. Mutational activation of PrfA, the transcriptional activator that induces the expression of LLO and the phospholipases that mediate vacuole membrane disruption, eliminates the need for pPplA, suggesting that pheromone signaling may be functionally linked to PrfA activation. To our knowledge, this is the first indication of a bacterial peptide pheromone being adapted to coordinate gene expression for an individual bacterium.

Interestingly, the escape defect associated with the loss of the pPplA pheromone appears thus far to be limited to non-professional phagocytic cells, as normal patterns of escape were observed in both primary macrophages as well as macrophage cell lines. Cell line-dependent differences in vacuole escape have been previously noted for *L*. *monocytogenes*. For example, bacterial escape from human epithelial cell lines does not require the activity of LLO, whereas escape is absolutely dependent upon this pore forming toxin in other cell types [65,66,68]. Rabinovich *et al* recently reported that gene products sharing homology with competence proteins involved in formation of the competence apparatus contribute to the escape of *L*. *monocytogenes* from professional phagocytes but not from non-professional phagocytic cells [90]. These observations indicate that the composition and/or properties of vacuolar membranes differ between different cell types, with the difference between professional phagocytes and non-professional phagocytic cells perhaps being most prominent. Based on their findings, Rabinovich *et*. *al* speculated that *L*. *monocytogenes com* genes may contribute to the formation of a pseudopilus structure that enhances phagosome lysis [90]. Bacterial competence and the formation of a Com-dependent psuedopilus are regulated by peptide pheromone-dependent signaling in other bacteria [91], however no peptide has thus far been associated with *L*. *monocytogenes com* gene expression. Our findings that the pPplA pheromone appears to enhance escape in non-professional phagocytic cells and not within professional phagocytes (where *com* genes are required) suggests that either the gene products that contribute to bacterial escape are differentially regulated by either pPplA or Com-associated regulation in specific cell types, or each system regulates a different set of gene products that contribute to escape within specific cell types. *L*. *monocytogenes* encodes gene products that share homology with those involved in Type IV secretion and the assembly of a conjugal DNA transfer system [92]; given the potential similarity of pPplA signaling pathways with those of cAD1 and Type IV secretion in *E*. *faecalis*, it is tempting to speculate that pPplA regulates the expression of a Type IV effector molecule and/or secretion apparatus that aides in vacuole escape.

An alternative hypothesis for the dependence of *L*. *monocytogenes* on pPplA expression is the possibility that the peptide itself enhances membrane lysis. Some bacterial cationic peptides have the ability to insert within lipid bilayers and disrupt membrane integrity [93]. While we have not yet been able to identify the precise composition of the pPplA peptide, it is likely to be hydrophobic in nature given the composition of the PplA signal sequence. We do not favor direct disruption of vacuole membranes by pPplA as we have not observed that the addition of pPplA-containing culture supernatants enhances LLO-dependent lysis of red blood cells for strains lacking *pplA*, or any indication that pPplA can disrupt membranes in the absence of LLO (S6 Fig.). Our data suggests that pPplA enhances vacuole escape through a mechanism that is not restricted to LLO activity as escape defects were still evident in Henle human epithelial cells, for which LLO is dispensable. Based on (1) the homology pPplA with cAD1 and its associated signaling components, and (2) the observation that pPplA induces the expression of a number of secreted gene products, we favor a model in which import of the pPplA pheromone stimulates the expression of *L*. *monocytogenes* gene products that enhance vacuole escape, possibly through the induction of a factor or factors that stabilize membrane perforations or pores.

Given that mutational activation of PrfA eliminates the requirement for pPplA-dependent signaling (Fig. 8), it is possible that pPplA contributes in some way to the process of PrfA activation. PrfA is postulated to require the binding of a small molecule cofactor for full activity, and pPplA could potentially be involved in: (1) the induction of a small molecule cofactor; (2) the induction of a transport system for import of the cofactor; or alternatively, (3) the induction of bacterial factors that stabilize or extend vacuole membrane perforations generated by LLO, allowing influx into the vacuole of a host-derived environmental signal. A role for pPplA in vacuole dissolution would be consistent with the fact that the pheromone is not required in strains containing constitutively activated PrfA*, as these strains secrete high levels of LLO and phospholipase which may be capable of mediating vacuole membrane disruption in the absence of any additional factors. While membrane perforation has been shown to occur rapidly within infected cells [60,74], the subsequent steps leading to complete membrane disruption and *L monocytogenes* cytosolic entry are less clearly defined. Finally, it should be noted that the PrfA cofactor binding pocket appears to have a high positive charge [94], thus we feel it is unlikely that the pPplA peptide itself serves as cofactor, although this remains a possibility.

Thus far, the principal *in vitro* phenotype we have identified in association with the secretion of the pPplA pheromone outside of cellular infection is the tendency for mutationally activated prfA* strains to aggregate in stationary phase static cultures (Fig. 3). While the relevance of this phenotype to *L*. *monocytogenes* physiology or virulence is not yet clear, bacterial aggregation has been associated with biofilm formation and virulence for a number of bacteria. In the case of *E*. *faecalis*, bacterial aggregation contributes to adhesion to mammalian host cells and the formation of large Enterococcal vegetative aggregates, consisting of a mix of bacteria, fibrin, platelets, and host cells in target organs such as the heart, where the bacterium causes infective endocarditis [95–99]. *E*. *faecalis* aggregation in culture has been used as a read out for the assessment of the synthesis and levels of the peptide-pheromones present in culture supernatants [47]. *E*. *faecalis* aggregation depends on the production of a surface protein known as the Aggregation substance (AS), and is required for the facilitation of conjugal DNA transfer of plasmids by bringing plasmid donor and recipient cells into close proximity for the formation of the conjugal bridge. *L*. *monocytogenes* is not known to contain a surface protein analogous to AS, however a recent study has reported that ActA, the surface protein required for host actin polymerization, promotes bacterial aggregation in broth culture in prfA* strains [50]. ActA-dependent bacterial aggregation was also associated with long term colonization of the intestines of mice. Interestingly, we have found that the aggregation of prfA* strains requires pPplA secretion in addition to surface expression of the ActA protein, as ΔpplA prfA* strains exhibited similar levels of ActA protein on their surface in comparison to prfA* strains (S2A Fig.), yet the ΔpplA prfA* strains were reduced for aggregation (Fig. 3B). Inhibition of protein translation through the addition of chloramphenicol to spent media containing pPplA resulted in reduced aggregation of ΔpplA prfA* strains (S7 Fig.), suggesting that translation is required in response to pheromone for efficient aggregation to occur. Finally, the reduced bacterial aggregation observed for prfA*Δ*pplA* strains is not due to any reduction in flagellar based swimming motility as this mutant exhibited the same swimming motility pattern on soft agar plates as the parental prfA* strain (S8 Fig.).

Given the important contributions of pPplA to *L*. *monocytogenes* pathogenesis, it would be useful to identify the amino acid composition of the pPplA peptide pheromone. To date, identification of the definitive amino acid sequence of pPplA has been extremely challenging. As described above, the only *in vitro* phenotype identified thus far in association with the pPplA pheromone is aggregation of prfA* strains in high density cultures grown in rich media. We have tried adding synthetic peptides based on the cAD1 sequence homology (ASSLLLVG) as well as the full length PplA signal sequence peptide to prfA* *ΔpplA* cultures to see if aggregation can be stimulated, however this approach has not been successful. It is possible that the *L*. *monocytogenes* pPplA peptide differs in length and/or composition in comparison to cAD1, and/or that the pPplA peptide is post-translationally modified. The precise amino acid composition and length of the active peptide of cAD1 is important for activity as the introduction of synthetic peptides containing additional N-terminal amino acids did not induce aggregation activity in *E*. *faecalis* [43]. Similarly, in *Streptococcus pyogenes* it has been recently demonstrated that single amino acid differences in peptide length or composition dramatically affect the activation of gene expression by Rgg proteins [100]. Peptide pheromones may be present and functional at very low concentrations (1 to 5 molecules of peptide are sufficient to stimulate the *E*. *faecalis* mating response [45,101]) and we are working to optimize a more sensitive assay that may enable us to purify pPplA from culture supernatants. In addition, there have been challenges associated with the cloning of *pplA* in *E*. *coli* plasmid vectors, suggesting that this gene may be somewhat toxic for *E*. *coli* host strains. Similar difficulties in cloning and overexpression of some of the Enterococcal peptide-pheromones have been reported [43,51] [102]. Purification of pPplA is likely to require the development of sensitive *in vitro* assays for the detection of peptide along with the optimization of the conditions for peptide expression.

**Fig 10:**
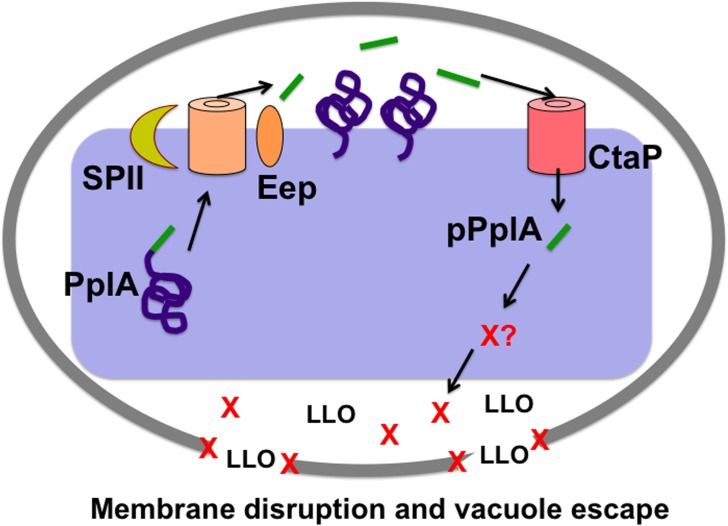
Model of *L*. *monocytogenes* pPplA signaling within the host vacuole. Modeling of the predicted *L*. *monocytogenes* pPlpA signaling pathway involved in enhancing vacuole escape from the host cell vacuoles in non-professional phagocytic cells. In wild-type *L*. *monocytogenes*, *pplA* encodes a lipoprotein (PplA) with a peptide pheromone (pPplA) located within the N terminal secretion signal peptide (shown in green). The signal sequence of prePplA is processed by signal peptidase II (SPII) and the released signal peptide is further cleaved by the protease Eep releasing the pPplA pheromone, while the PplA protein becomes lipid modified and associated with the membrane or secreted. Upon entry of wild-type *L*. *monocytogenes* into non-professional phagocytic host cell, the confined space of the vacuole leads to import of the secreted pPplA pheromone through the CtaP peptide transporter. pPplA accumulation in the bacterial cytoplasm stimulates a signaling cascade that results in the production of an unknown factor (X) that contributes to vacuole lysis. Factor X may function by helping to stabilize the LLO generated membrane pore, facilitating eventual vacuole membrane dissolution as well as the influx of mammalian cytosol components that may promote PrfA activation and the expression of gene products required for intracellular growth and cell-to-cell spread. In the absence of pPplA (Δ*pplA* strains), bacterial escape is delayed until sufficient LLO and phospholipase accumulate to disrupt the vacuole membrane in the absence of factor X function. For strains containing constitutively activated PrfA*, the substantially increased secretion of LLO and the phospholipases is sufficient to disrupt the vacuole membrane in the absence of factor X.

In summary, we have described a novel peptide-based signaling system in *L*. *monocytogenes* that is required for bacterial virulence. The pPplA peptide pheromone, processed from the N-terminal secretion signal sequence of *pplA*, has functionally evolved to enhance vacuolar escape in non-professional phagocytic cells. Based on data presented here, we propose the following as a working model for the role of pPplA in *L*. *monocytogenes* pathogenesis (Fig. 10): *L*. *monocytogenes* produces a basal level of PplA that is secreted through bacterial cellular membrane by the general secretory pathway. Following secretion, the N-terminal secretion signal peptide of PplA gets cleaved by Signal Peptidase II releasing the lipoprotein of PplA, which is modified with diacyl glycerol and anchored to the cellular membrane by Lgt [103]. The membrane embedded signal peptide is further processed by Eep and possibly other proteases to release the mature pPplA peptide into the extracellular space. Once *L*. *monocytogenes* enters a host cell and is contained within a vacuole, the pPplA peptide accumulates in the confines of the vacuolar space and is transported back into the bacterial cell through the CtaP transport system, thus initiating a signaling cascade to induce the expression of gene products that contribute to vacuolar escape. These gene products may serve to stabilize membrane pores induced by LLO and phospholipases, facilitating the influx into the vacuole of a host-derived environmental signal that stimulates PrfA activation. Fully activated PrfA induces the expression of additional LLO and phospholipase as well as gene products required for bacterial replication within the host cytosol and for spread to adjacent host cells. While much remains to be elucidated about this intriguing pheromone signaling pathway, it serves to illustrate the diversity of cellular processes regulated by small peptides in Gram positive bacteria.

## Materials and Methods

### Bacterial strains, plasmids and growth conditions

*L*. *monocytogenes* and *E*. *coli* strains used in this study are listed in Table 2. *E*. *coli* XL1-Blue (Agilent Technologies, Santa Clara, CA), One Shot TOP10 (Invitrogen Corp., Carlsbad, CA), NEB 5αF’*I* ^*q*^ (New England Biolabs, Ipswich, MA), SM10s, S17-1 and BH10s (both kind gifts of Nicholas Cianciotto, Northwestern University) were used as host strains for maintenance and propagation of recombinant plasmids. *L*. *monocytogenes* and *E*. *coli* strains were grown at 37°C in brain heart infusion (BHI) media (Difco Laboratories, Detroit, MI) and Luria broth (LB) (Invitrogen Corp., Carlsbad, CA). Maintenance of the integration plasmid pPL2 was selected for using 25 μg/ml of chloramphenicol in *E*. *coli* and 5 μg/ml in *L*. *monocytogenes*. Maintenance of the integration plasmid pIMK2 (a kind gift of Dr. Colin Hill, University College Cork) was selected for using 50 μg/ml of kanamycin in *E*. *coli* and in *L*. *monocytogenes*. Bacteria containing the 6X Histidine tagged expression vector pQE30 (Qiagen Inc., Valencia, CA) were maintained in *E*. *coli* with 100 μg/ml ampicillin. Streptomycin 200 μg/ml was used in selection of *L*. *monocytogenes* following bacterial conjugation and isolation from organs of infected mice.

**Table 2:** Bacterial strains and plasmids used in this study.

Strain	Description	Source/Reference
***L*. *monocytogenes***		
NF-L100	Wild type 10403S	[115]
NF-L340	Δ*hly* strain (DP-L2161)	[116]
NF-L1166	prfA*L140F	[117]
NF-L1553	Δ*ctaP* (*lmo0135*)	[58]
NF-L1678	NF-L1166 (prfA* L140F) Δ*ctaP*	This study
NF-L3069	*oppA*-gene disruption	This study
NF-L3080	pNF-1002 (prfA* L140F) integrated into NF-L3069	This study
NF-L3095	Δ*lmo1318 (eep)*	This study
NF-L3101	*pplA*-G72stop codon mutant	This study
NF-L3137	Δ*lmo2637 (pplA)*	This study
NF-L3150	NF-L1166 (prfA*L140F) Δ*pplA*	This study
NF-L3178	pNF-1002 (prfA* L140F) integrated into NF-L3095 (Δ*eep*)	This study
NF-L3180	pNF-1002 (prfA* L140F) integrated into NF-L3101 (*pplA*-G72stop)	This study
NF-L3510	pNF-3184 integrated into NF-L3137 (Δ*pplA*), complementation with entire *pplA* ORF	This study
NF-L3512	pNF-3185 integrated into NF-L3137 (Δ*pplA*), complementation with first 72 amino acids of *pplA*	This study
***E*. *coli***		
XL1-Blue	*E*. *coli* propagation strain	Agilent Technologies
NEB 5αF’I^*q*^	*E*. *coli* protein expression strain	NEB
TOP10	*E*. *coli* propagation strain	Invitrogen
BH10	*E*. *coli* plasmid copy number restrictive propagation strain	
SM10	*E*. *coli* conjugation strain	
S17-1	*E*. *coli* conjugation strain	
**Plasmids**		
pQE30	N-terminal His-tagged expression vector	Qiagen
pKSV7	Temperature-sensitive *L*. *monocytogenes* allelic exchange vector	[104]
pIMK2	pPL2 derived complementation integration vector	[35]
pNF-1002	pPL2 site specific integration vector with full length prfA*L140F and all promoters	[37]
pNF-3066	pKSV7 plus 500 base pair internal fragment of *oppA*	This study
pNF-3077	pKSV7 plus *pplA*-G72_STOP_ codon construct	This study
pNF-3086	pKSV7 plus Δ*lmo1318 (eep)* construct	This study
pNF-3102	pKSV7 plus Δ*lmo2637 (pplA)* construct	This study
pNF-3184	pIMK2 plus *pplA* entire coding region	This study
pNF-3185	pIMK2 plus first 72 amino acids of *pplA*	This study
pNF-3359	pQE30 plus *pplA* lipoprotein from amino acid 72 to stop codon	This study

### Plasmid and bacterial mutant construction

Primer pairs used for construction of in-frame deletions of *lmo2637 (pplA)* and *lmo1318 (eep)*, introduction of a premature stop codon at amino acid G72 of *pplA*, and complementation of the *pplA* deletion mutant are listed in Table 3. Mutants were generated by cloning 600 base pairs of the immediate upstream and downstream regions of their respective coding regions into the temperature-sensitive shuttle plasmid pKSV7 [104], leaving only the translational start and stop of the open reading frames. Due to apparent difficulties in the cloning of DNA sequences toxic to *E*. *coli*, only 250 base pairs of the flanking regions of *pplA* were amplified for construction of the *pplA* deletion mutant vector construct. The flanking regions were joined using SOEing PCR, and the SOEing PCR products were digested with the appropriate restriction enzymes and ligated into pKSV7 to generate the mutant constructs. The *ermB* gene encoding erythromycin (Em) resistance and containing its native promoter was PCR amplified from pHY-304 (a kind gift from Dr. Craig Rubens and Dr. Amanda Jones, Seattle Children’s Hospital Research Foundation) and inserted in between the internal Kpn*I* sites of the flanking regions in the pKSV7-based *pplA* deletion construct. All pKSV7-*pplA* containing plasmids had to be maintained in the *E*. *coli* host restrictive strain BH10s to maintain stable plasmid constructs. Gene-disruption mutants were constructed by amplification of a 500 base pair internal fragment of the appropriate coding region that was then ligated into pKSV7. The resulting plasmid constructs were transformed into the *L*. *monocytogenes* wild-type strain 10403S. *pplA* deletion mutants were selected based on Em^R^ and Cm^S^, and confirmed by PCR amplification of products from *L*. *monocytogenes* chromosomal DNA. *eep* and *pplA*-G72stop mutants were screened for Cm^S^ and confirmed by PCR for the *eep* deletion mutant, and sequencing for the *pplA*-G72stop codon mutant. *oppA* gene disruption mutants were maintained by selecting for Cm^R^.

**Table 3:** Oligonucleotides used in this study.

Primer	Sequence (5’→3’)^*a*^ ^,^ ^*b*^	Reference
*pplA*-*S*oeA	GC*TCTAGA*CCTCCTACTAATGTCTAGAT	This study
*pplA*-SoeB	AAATTGTGCTAATTA*GGTACC*CATGTTTTGCTCCCA	This study
*pplA*-SoeC	TGGGAGCAAAACATG*GGTACC*TAATTAGCACAATTT	This study
*pplA*-SoeD	CG*GAATTC*GCCCATTAGAAAGTCTG	This study
*pplA*-MutF	GC*TCTAGA*CACCTGCATATCCTG	This study
G72mut-B	TTCGATTGACATGAA**TTA**TTTCCAGCCTTTGTC	This study
G72mut-C	GACAAAGGCTGGAAA**TAA**TTCATGTCAATCGAA	This study
*pplA*-MutR	CG*GAATTC*AACAAGAGAGATTTGTTAAG	This study
*eep*-SoeA	AAAA*CTGCAG*ACAAGCATTAAACGGA	This study
*eep*-*S*oeB	GTCTATCTCTTTTTA*GGTACC*CAAAATAGCTTCACC	This study
*eep*-*S*oeC	GGTGAAGCTATTTTG*GGTACC*TAAAAAGAGATAGAC	This study
*eep*-SoeD	TCC*GAGCTC*TAGCAGCATAATCAGAAG	This study
*oppA*-insF	GC*GGTACC*CTCACTATTCGCATTC	This study
*oppA*-insR	GC*GGTACC*GTTTTGCAGTTTAGAT	This study
pIMK2C’-F	ATAT*CCATGG*AATTGAAAAAGTAGCAAT	This study
pIMK2C’-R	ATAT*CCCGGG*TTATTCAGCTTCTACTAGTT	This study
*pplA*His-F	CGC*GGATCC*GGTTTCATGTCAATCGAAGTT	This study
*pplA*His-R	TCC*CCCGGG*TTATTCAGCTTCTACTAGGTT	This study

^*a*^Italicized letters indicate restriction enzymes used in making constructs. Primer pairs used for construction of an in-frame *pplA* deletion mutant were *pplA-S*oeA-*XbaI* and *pplA-S*oeB to amplify the upstream flanking region and *pplA*-SoeC and *pplA*-SoeD-*EcoRI* to amplify the downstream flanking region, both *pplA*-SoeB and *pplA*-SoeC contain internal *KpnI* sites for insertion of the *ermB* gene (similar combination of primer pairs were used for construction of the respective *pplA*-G72 stop codon mutant and the *eep* deletion mutant, but *eep*-SoeA contained a *PstI* site and *eep*-SoeD contained a *SacI* site). *oppA* gene-disruption primers contained a *KpnI* site, pIMK2C’F a *NcoI* site, pIMK2C’R a *XmaI* site, *pplA*His-F a *KpnI* site, and *pplA*His-R an *XmaI* site.

^*b*^Letters in bold indicate the premature stop codon engineered at amino acid position G72 in the *pplA* coding sequence.

The integration plasmid, pIMK2, was used for complementation of the *pplA* deletion mutant. This plasmid is a derivative of the conjugative plasmid, pPL2, and integrates in single copy into the *L*. *monocytogenes* 10403S chromosome at a phage attachment site within the tRNA^Arg^ gene following conjugation [35]. For construction of the complementation vectors, the entire open reading of *pplA* beginning with the second codon and ending with the stop codon or just up to the first 72 amino acids were PCR amplified and digested with Nco*I* and Xma*I* and subsequently ligated together with pIMK2 vector and transformed into *E*. *coli* XL1-Blues. The resulting construct was initially electroporated into the wild-type background strain using the method as described by Monk *et al*., resulting in a strain containing two copies of *pplA*. Tranduction of the Δ*pplA*::*erm* mutation into this pIMK2-*pplA* integrated strain or different background strains was performed as previously described [33,105]. pPL2-derived plasmids were conjugated into the various *L*. *monocytogenes* strains.

For generation of purified truncated PplA protein used for antibody affinity purification and as a control standard for Western blot analyses, the lipoprotein region of *pplA* from the G72 amino acid position to the stop codon was PCR amplified from *L*. *monocytogenes* strain 10403S (wild-type) using the primer pairs listed in Table 3 and subsequently cloned into a pQE30 Expression vector (Qiagen Inc., Valencia, CA), which contains an N-terminal 6x-Histidine tag and an isoproyl-β-D-thiogalactopyranoside (IPTG) inducible promoter. The resulting construct was initially propagated in *E*. *coli* TOP10 cells, isolated and then transformed into NEB 5αF’*I* ^*q*^ (New England Biolabs, Ipswich, MA). An overnight culture containing the expression construct was diluted 1:50 in fresh LB broth and the culture was incubated at 37°C (with shaking) until an optimal density of 0.5 was reached. To induce expression of the PplA protein, 1 mM IPTG (Inalco, Paris, France) was added to the culture and induction was allowed to proceed for 3 to 4 hours. The bacterial cells were recovered by centrifugation, resuspended in 1 ml of PBS and 20 mg of lyzozyme (Sigma-Aldritch, St. Louis, MO), and incubated at 37°C for 30 minutes. Prior to sonication with 5 repeated 10 second bursts and 1 minute cooling on ice, 10 ul of 100X protease inhibitor cocktail was added. The soluble fraction containing the N-His-PplA protein was collected and purified using the His-Pur Purification Kit (Thermo Scientific, Rockford, IL). Protein concentration was determined using a BCA Protein Assay Kit (Thermo Scientific; Rockford, IL).

### Western blot analysis of PplA lipoprotein

PplA lipoprotein was detected from both secreted protein preparations isolated from culture supernatants and surface-associated fractions isolated from bacterial whole cells as previously described with slight modifications [38,106,107]. In brief, 100 ml culture of each *L*. *monocytogenes* strain was grown to mid-log and stationary phase in BHI at 37°C with shaking. Samples were all normalized to equivalent cell densities (OD_600_ nm). Proteins present in the culture supernatants were precipitated with Trichloroacetic acid (TCA, Fisher Scientific, Hanover Park, IL), and pellets were resuspended in 100μl of 2X SDS-boiling buffer (Bio-Rad, Hercules, CA). Surface-associated (SA) proteins were extracted by boiling of the bacterial pellet in 200 μl of 2X SDS-boiling buffer (Bio-Rad, Hercules, CA). For detection of PplA lipprotein, 10 μl of the isolated secreted proteins sample and 20 μl of SA proteins were separated using SDS-polyacrylamide gel electrophoresis. Protein samples were transferred onto PVDF membranes. PplA lipoprotein was detected using a 1:200 dilution of a affinity-purified polyclonal antibody directed against the C-terminal of the PplA lipoprotein (FKIYAAQLQN), in 1X PBST (Phosphate buffered saline solution plus 0.05% Tween-20) followed by incubation with a 1:2500 dilution of a polyclonal Goat-anti rabbit secondary antibody conjugated to alkaline-phosphatase (SouthernBiotech, Birmingham, AL). Bands were visualized colormetrically with the addition of 10ml of a BCIP/NBT Plus solution (SouthernBiotech, Birmingham, AL). Densitometry was determined using ImageJ software (http://rsbweb.nih.gov/ij/download.html).

### Bacterial aggregation assay

Three mls of various *L*. *monocytogenes* strains were grown in BHI at 37°C with shaking overnight. One ml of the overnight stationary phase culture was removed and left static at room-temperature and the optical-density at 600nm was measured at the indicated time points. Bacterial aggregation was indicated via the evident decrease of the optical-density of the culture supernatant as the bacteria aggregated out of solution.

### Mouse infections

All animal procedures were IACUC approved and performed in the Biological Resources Laboratory at the University of Illinois at Chicago. Overnight bacterial overnight cultures were diluted 1:20 into fresh media and grown to an OD_600_ ∼0.6. 1 ml of culture (corresponding to 6x10^8^ CFU/ml) was washed, diluted and resuspended in PBS to a final concentration of 1x10^5^ CFU/ml. 8–10 week old female Swiss Webster mice (Charles River Laboratories, Chicago, IL) were injected with 200 ul PBS containing 2x10^4^ CFU *L*. *monocytogenes* via the tail vein. At 24, 48 and 72 hrs post-infection, mice were sacrificed and livers and spleens were harvested. Organs were homogenized with a Tissue Master 125 homogenizer (Omni International, Kennesaw, GA) and dilutions were plated onto BHI streptomycin (200 ug/ml) plates. Non-paired student *t*-test was used for statistical analysis.

### Bacterial intracellular growth assays

Bacterial intracellular growth assays in Potoroo tridactylis kidney epithelial cells (PtK2) were performed as previously described [37,68,108]. In brief, monolayers of cells were grown on glass coverslips to confluency and infected with bacterial strains with an MOI of 100:1. One hour post-infection, monolayers were washed 3X in PBS and 5 μg/ml of gentamicin was added to kill extracellular bacteria. At indicated time points, coverslips were removed and lysed in 5 mls of sterile H_2_O to release intracellular bacteria for enumeration of intracellular growth or were processed for microscopy. Actin localization studies were done as previously described [31] except that host cell actin was stained with Alexa Fluor 488 phalloidin (Invitrogen Corp.) at 2, 4, and 6 hours p.i.

For staining of host cell Rab7, PtK2 cells grown on glass coverslips were infected with bacteria as described above. Coverslips were removed at 1.5 hours post-infection, and cells were fixed and permeabilized as previously described for host F-Actin staining but with phalloidin conjugated to Alexa-350. Coverslips were then blocked for 30 minutes in PBS with 10% FBS, washed 5 times in PBS, stained with goat anti-Rab7 (Santa Cruz Biotechnology) for 30 minutes at RT, washed 5 times in PBS, followed by staining with donkey anti-goat conjugated to Texas Red (Santa Cruz Biotechnology) for 30 minutes at RT. *Listeria* in the infected cells was stained with a rabbit anti-Listeria (BD Biosciences) for 30 minutes at RT, washed in PBS, followed by incubation with a goat anti-rabbit secondary antibody conjugated to Alexa-488 (Invitrogen Corp.) for 30 minutes at RT.

For detection of bacteria contained in vacuoles by binding with YFP-CBD (yellow fluorescent protein fused to the cell wall binding domain of the phage endolysin Ply118, which binds to the *Listeria* cell surface) [74], semi-confluent monolayers of PtK2 cells grown in 24 well plates were transfected with the YFP-CBD plasmid (a kind gift from Joel A. Swanson, University of Michigan, Ann Arbor, MI) using Fugene 6 (Promega) as per manufactures protocol. Transfected cells were then infected with *L*. *monocytogenes* and at 15 and 30 minutes post-infection, coverslips were removed and processed for microscopy. Host cell F-actin was stained using phalloidin conjugated to Texas Red and DNA was stained with DAPI. Bacteria bound with YFP-CBD appeared green.

For infection of murine bone-marrow derived macrophages (BMMØ), macrophages were isolated from the femurs of mice and maintained as described [109] and intracellular growth was performed with an MOI of 0.1:1 as described above for J774 cells. Where indicated, 1 ηg/ml of recombinant mouse IFN-γ(Invitrogen Corp., Carlsbad, CA) was added to monolayers of BMMØ 24 hours prior to infection to activate the macrophages.

### Measurement of bacterial cell-to-cell spread

Plaque assays were conducted as previously described [110]. Briefly, murine L2 fibroblasts were grown to confluency in 6-well microtiter plates and infected with 20 μl of a normalized 1:20 dilution of overnight culture grown at 37°C in BHI with shaking (MOI 10:1). One hour post-infection, L2 infected monolayers were washed and 10 μg/ml of gentamicin was added to kill extracellular bacteria. Three days post-infection, Neutral Red (Sigma-Aldrich, St. Louis, MO) was added and plaques were visualized and measured using a micrometer (Finescale, Orange County, CA).

### Measurement of β-glucuronidase activity

Overnight cultures of *L*. *monocytogenes* were diluted 1:20 in fresh BHI or LB containing 25 mM glucose-6-phosphate (Sigma-Aldrich, St. Louis, MO) and 0.2% activated charcoal and grown with shaking at 37°C. At various time points, the OD_600_ was determined for each culture and 1 ml of each sample was centrifuged. Bacterial pellets were resuspended in 1 ml of ABT buffer [1M potassium phosphate (pH 7.0), 0.1 M NaCl, 1% Triton] and ß-Glucuronidase (GUS) activity was measured as described by Youngman [111] with the substitution of 4-methylumbelliferyl-ß-D-glucuronided (Sigma-Aldrich, St. Louis, MO) in place of 4-methylumbelliferyl-ß-D-galactoside.

### Assessment of PlcB-associated phospholipase activity

*plcB*-dependent phospholipase production was assayed on egg yolk agar plates [66,112]. Antibiotic-free chicken egg yolk was added in a 1:1 (vol/vol) ratio to PBS and vortexed to form a suspension. 5 ml of egg yolk suspension was added to 100 ml of molten LB medium plus 0.2% activated charcoal (Sigma-Aldrich, St. Louis, MO) and 25mM glucose-6-phosphate (Sigma-Aldrich, St. Louis, MO), [68,113] and 10 ml of this mixture was poured into Petri dishes. Bacterial strains were gently streaked onto the surface of the plate and incubated at 37°C for 24h. Phospholipase activity was visualized as a zone of opacity surrounding bacterial streaks.

### Measurement of LLO-associated hemolytic activity

Stationary-phase bacterial cultures were diluted 1:10 into LB medium and grown at 37°C for 5h with shaking. Optical density OD_600_ was determined, and 1 ml of each culture was normalized and centrifuged at 13,000 x g for 5 min. The supernatant was collected and was assayed for LLO-associated hemolytic activity using phosphate-buffered saline (PBS)-washed sheep erythrocytes (Cocalico Biologicals Inc., Reamstown, PA) as previously described [114]. Hemolytic activity was determined as the reciprocal of the supernatant dilution at which 50% lysis of erythrocytes was observed.

The amount of secreted LLO protein present in the culture supernatants was detected as previously described [68].

### 2-Dimensional gel electrophoresis and mass spectrometry

Isolation of *L*. *monocytogenes* secreted proteins, 2-dimensional gel electrophoresis (Kendrick Labs, Madison, WI), and identification of protein spot differences (Proteomics Core Facility, Research Resources Center at the University of Illinois at Chicago) were all processed as previously described with one minor modification [68,107]. Proteins isolated for this study were obtained from stationary phase cultures grown overnight versus exponential phase cultures that were previously assessed.

### Membrane integrity assessments

Membrane permeability differences between the varying strains were assessed using the LIVE/DEAD *Bac*Light Bacterial Viability Kit (Invitrogen Corp., Carlsbad, CA) as previously described [58].

## Supporting Information

S1 Fig Coomassie stain and Western analysis of *L*. *monocytogenes* secreted and cell surface-associated proteins following SDS-PAGE. **(A)** *L*. *monocytogenes* strains were grown to stationary phase in BHI at 37°C with shaking overnight. Samples were normalized to optical-density 600nm of 1.5. Bacteria were recovered and non-covalently associated surface proteins were extracted from the bacterial pellets by boiling in SDS-boiling buffer. Secreted proteins present in the culture supernatants were TCA-precipitated and the isolated protein pellet was resuspended in SDS-boiling buffer. Protein samples were then separated by SDS-PAGE and proteins were visualized by coomassie staining. **(B)** Western blot analysis of the PplA lipoprotein of samples isolated as described in panel A from wild-type *L*. *monocytogenes*, the *pplA* ^*m*^ mutant, which contains three amino acid substitutions in the predicted pPplA region within the chromosome, and the Δ*ctaP* oligopeptide transport mutant. The His-purified truncated C-terminal region of the PplA lipoprotein was included as the positive control. The arrow indicates the full length lipoprotein.
(PDF)

S2 Fig Reduced bacterial aggregation in a prfA* *ΔpplA* is not due to decreased levels of ActA protein, and treatment of spent media with protease K eliminates bacterial aggregation. **(A)** Measurement of bacterial surface-associated ActA protein levels. Overnight cultures of the various strains were grown overnight to stationary phase in BHI at 37°C with shaking, cells were normalized to optical-density 600nm, centrifuged, and non-covalently associated cell surface proteins were extracted by boiling in SDS-boiling buffer. The presence of ActA was detected using western blot analysis with antibodies directed against ActA. **(B)** Assessment of bacterial aggregation in spent media derived from the *pplA*-G72_STOP_ codon mutant cultures with and without proteinase K treatment. Prior to measurement of bacterial aggregation, a portion of spent media derived from *pplA*-G72_STOP_ was treated with 50 μg/mL of proteinase K for 30 minutes at 37°C, and the protease was heat-inactivated at 65°C. *L*. *monocytogenes* strains assayed for bacterial aggregation were recovered and resuspended in spent media treated or untreated with proteinase K, and the optical-density 600nm was measured over time. For both panels A and B, data is representative of at least two-independent experiments.
(PDF)

S3 Fig Loss of pPplA impairs the ability of *L*. *monocytogenes* to form plaques in cell monolayers. **(A)** The ability of the various *L*. *monocytogenes* strains to invade, multiply, and spread from cell-to-cell was determined by assessing plaque formation within monolayers of fibroblast tissue culture cells. Cells were infected with an MOI of 10:1 and at 1 hour post-infection were washed, gentamicin was added, and plaques were visualized three days post-infection by staining with Neutral Red. Zones of clearing that did not stain indicate plaque formation. **(B)** Quantification of the diameter of plaques formed compared to wild-type size (set to 100%). At least 20 plaques from three independent experiments were counted and measured for each strain. Data shown for panels A and B are representative of three independent experiments done in duplicate. Loss of *pplA* but not the lipoprotein (*pplA*-G72stop mutant) impaired the ability of *L*. *monocytogenes* to reach the host cytosol and spread efficiently from cell-to-cell.
(PDF)

S4 Fig Δ*pplA* mutant and wild-type *L*. *monocytogenes* infection of IFNγ-treated bone marrow-derived macrophages (BMMØ). Measurement of intracellular growth of wild-type and Δ*pplA* mutant in BMMØ using an MOI of 0.1:1. Macrophages were treated with 1 ng/mL IFNγ twenty-four hours prior to bacterial infection. Data shown is representative of three-independent experiments.
(PDF)

S5 Fig *L*. monocytogenes prfA*, prfA* Δ*pplA* and prfA* *pplA*-G72_STOP_ mutants display similar secreted protein profiles, and mutant bacteria do not exhibit increased cell lysis or decreased membrane integrity. **(A)** Two-dimensional gel electrophoresis of secreted proteins overnight to stationary phase cultures grown in BHI. Secreted proteins were TCA precipitated from bacterial culture supernatants and processed for 2-D gel analysis. **B)** Measurement of membrane integrity of bacterial strains grown in BHI with shaking overnight at 37°C to stationary phase. Bacterial cells were normalized to optical-density 600nm of 1.5, and cells were diluted 1:10 in PBS and stained with the LIVE/DEAD Bac*Light* Viability Kit as per manufacture’s direction. Live bacterial cells with intact membranes fluoresce green due to the uptake of the membrane permeant SYTO9 dye, and dead cells or cells with compromised membranes incorporate the membrane impermeant propidium iodide (PI) dye and stain red. A minimum of 10 fields from two-independent experiments were visualized.
(PDF)

S6 Fig pPlpA containing culture supernatants do not enhance LLO-dependent lysis of sheep red blood cells. Measurement of LLO-associated hemolytic activity as assessed by lysis of sheep red blood cells from serial dilutions of mixed culture supernatants of bacterial strains grown shaking in LB for 5 hours at 37°C. Hemolytic activity was determined as the reciprocal of supernatant dilution at which 50% lysis was observed and the data is reported as the percentage of WT, with WT values set to 100%. Assays were carried out using a 1:1 ratio of mixed culture supernatants to determine if supernatants derived from a Δ*hly* mutant, which does not produce secreted LLO but still produces the pPplA peptide, could directly enhance lysis of RBC when added to supernatants derived from Δ*pplA* mutant, which does not contain peptide but contains LLO. Lysis activity was compared to Δ*pplA* culture supernatants mixed with supernatants from a Δ*hly* Δ*pplA* double mutant (no secreted LLO or peptide). No enhancement of cell lysis was observed when supernatants containing peptide but no LLO were mixed with supernatants containing LLO but no peptide. Data is representative of three independent experiments.
(PDF)

S7 Fig Bacterial aggregation is reduced when protein synthesis is inhibited. Assessment of bacterial aggregation in the presence of the antibiotic chloramphenicol, an inhibitor of protein translation. The optical-density at 600nm was monitored at the indicated points for wild-type, prfA*, or prfA* Δ*pplA* strains of stationary-phase bacteria resuspended in 1 mL of spent media derived from *pplA*-G72_STOP_ cultures with or without the addition of 10 μg/mL of chloramphenicol during static incubation. Data shown is representative of at least two-independent experiments.
(PDF)

S8 Fig Loss of pPplA does not impair flagellar swimming motility in a prfA* background strain. Swimming motility was assessed on semisolid (0.3% w/v agar) BHI media. Plates were inoculated with 5 μL of the indicated *L*. *monocytogenes* strains grown overnight to stationary phase in BHI with shaking and normalized to an optical-density 600nm of 1.5. The inoculated plates were then incubated overnight at 37°C. Swimming motility is evident from the migration of the bacteria away from the original spot of inoculation. Data shown is representative of at least three-independent experiments.
(PDF)
