# Supplementary material for: Identification of a Peptide-Pheromone that Enhances Listeria monocytogenes Escape from Host Cell Vacuoles
Source: PLoS Pathog. 2015 Mar 30;11(3):e1004707. doi: 10.1371/journal.ppat.1004707 (PMC4379056; doi:10.1371/journal.ppat.1004707)
Supplement: S1 Fig — (A) L. monocytogenes strains were grown to stationary phase in BHI at 37°C with shaking overnight. Samples were normalized to optical-density 600nm of 1.5. Bacteria were recovered and non-covalently associated surface proteins were extracted from the bacterial pellets by boiling in SDS-boiling buffer. Secreted proteins present in the culture supernatants were TCA-precipitated and the isolated protein pellet was resuspended in SDS-boiling buffer. Protein samples were then separated by SDS-PAGE and proteins were visualized by coomassie staining. (B) Western blot analysis of the PplA lipoprotein of samples isolated as described in panel A from wild-type L. monocytogenes, the pplA m mutant, which contains three amino acid substitutions in the predicted pPplA region within the chromosome, and the ΔctaP oligopeptide transport mutant. The His-purified truncated C-terminal region of the PplA lipoprotein was included as the positive control. The arrow indicates the full length lipoprotein. (PDF) [file ppat.1004707.s001.pdf]

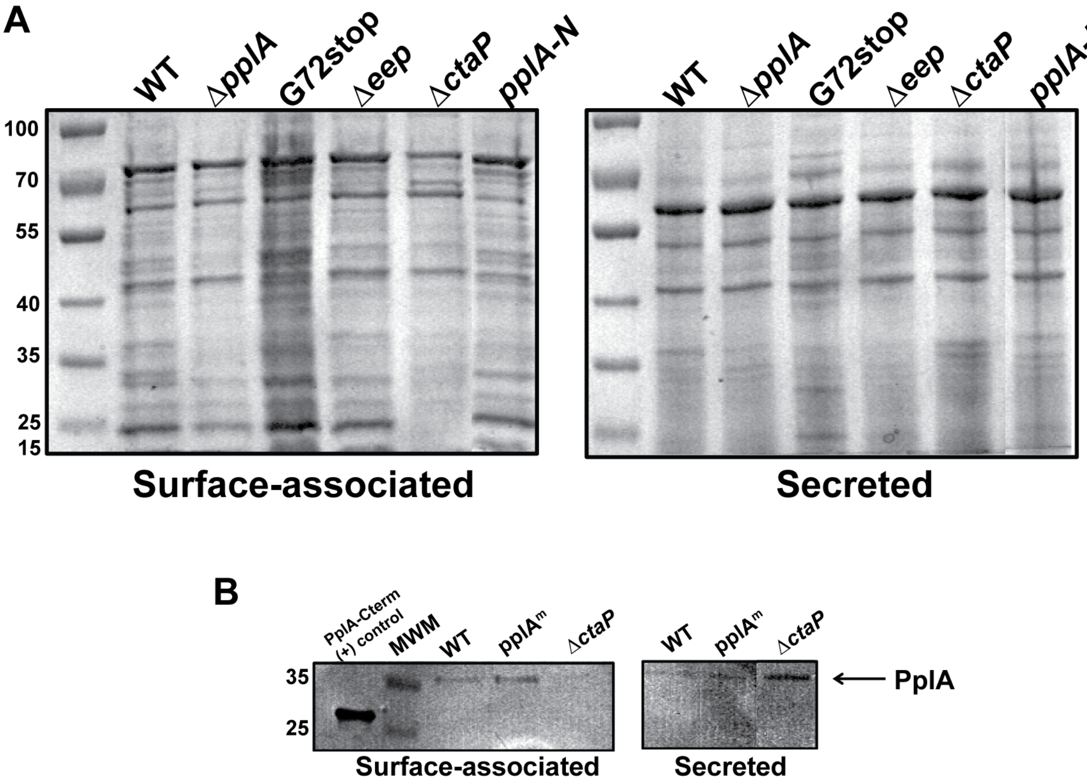

Supplemental Figure S1. Coomassie stain and Western analysis of *L. monocytogenes* secreted and cell surface-associated proteins following SDS-PAGE. **(A)** *L. monocytogenes* strains were grown to stationary phase in BHI at 37°C with shaking overnight. Samples were normalized to an optical density at 600nm of 1.5. Bacteria were recovered and non-covalently associated surface proteins were extracted from the bacterial pellets by boiling in SDS-boiling buffer. Secreted proteins present in the culture supernatants were TCA-precipitated and the isolated protein pellets were resuspended in SDS-boiling buffer. Protein samples were separated using SDS-PAGE and proteins were visualized by coomassie staining. **(B)** Western blot analysis of the PplA lipoprotein of samples isolated as described in panel A from wild-type *L. monocytogenes*, the $pplA^{m}$ mutant, which contains three amino acid substitutions within the predicted pPplA peptide region within the chromosome, and the Δ*ctaP* oligopeptide transport mutant. The His-purified truncated C-terminal region of the PplA lipoprotein was included as the positive control. The arrow indicates the full length PplA lipoprotein.
