# Supplementary material for: Identification of a Peptide-Pheromone that Enhances Listeria monocytogenes Escape from Host Cell Vacuoles
Source: PLoS Pathog. 2015 Mar 30;11(3):e1004707. doi: 10.1371/journal.ppat.1004707 (PMC4379056; doi:10.1371/journal.ppat.1004707)
Supplement: S2 Fig — (A) Measurement of bacterial surface-associated ActA protein levels. Overnight cultures of the various strains were grown overnight to stationary phase in BHI at 37°C with shaking, cells were normalized to optical-density 600nm, centrifuged, and non-covalently associated cell surface proteins were extracted by boiling in SDS-boiling buffer. The presence of ActA was detected using western blot analysis with antibodies directed against ActA. (B) Assessment of bacterial aggregation in spent media derived from the pplA-G72STOP codon mutant cultures with and without proteinase K treatment. Prior to measurement of bacterial aggregation, a portion of spent media derived from pplA-G72STOP was treated with 50 μg/mL of proteinase K for 30 minutes at 37°C, and the protease was heat-inactivated at 65°C. L. monocytogenes strains assayed for bacterial aggregation were recovered and resuspended in spent media treated or untreated with proteinase K, and the optical-density 600nm was measured over time. For both panels A and B, data is representative of at least two-independent experiments. (PDF) [file ppat.1004707.s002.pdf]

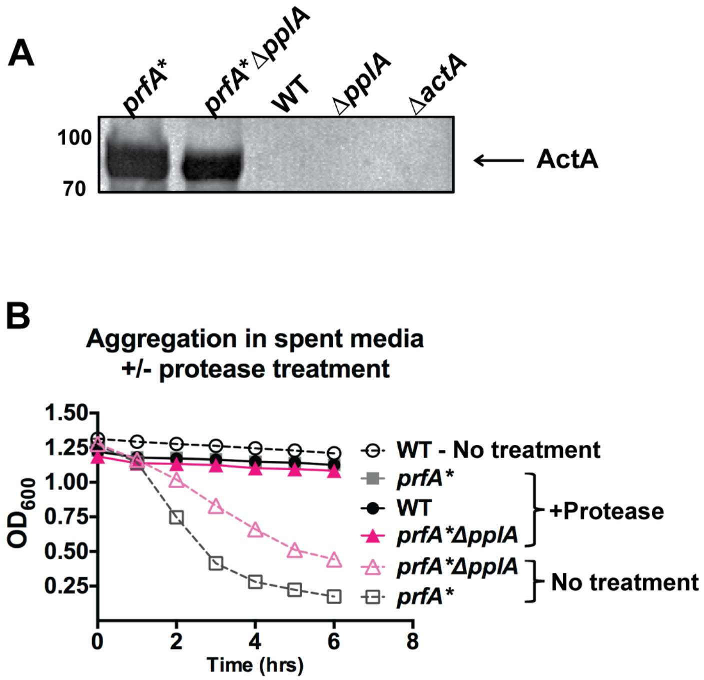

Supplemental Figure S2. Reduced bacterial aggregation in a *prfAΔ*pplA* is not due to decreased levels of ActA protein, and treatment of spent media with protease K eliminates bacterial aggregation. (A)** Measurement of bacterial surface-associated ActA protein levels. Overnight cultures of the indicated strains were grown overnight to stationary phase in BHI at 37°C with shaking, cells were normalized to an optical density at 600nm of 1.5, and non-covalently associated cell surface proteins were extracted by boiling recovered bacteria in SDS-boiling buffer. The presence of ActA was detected using western blot analysis with antibodies directed against ActA. **(B)** Assessment of bacterial aggregation for bacteria incubated in spent media derived from *pplA*-$G72_{STOP}$ codon mutant cultures with and without proteinase K treatment. Prior to measurement of bacterial aggregation, a portion of spent media derived from *pplA*-$G72_{STOP}$ cultures was treated with 50 μg/mL of proteinase K for 30 minutes at 37°C, then protease was heat-inactivated at 65°C. *L. monocytogenes* strains to be assayed for bacterial aggregation were recovered and resuspended in spent media treated or untreated with proteinase K, and the optical-density 600nm was measured over time. For both panels (A) and (B), data is representative of at least two-independent experiments.
