# Supplementary material for: Identification of a Peptide-Pheromone that Enhances Listeria monocytogenes Escape from Host Cell Vacuoles
Source: PLoS Pathog. 2015 Mar 30;11(3):e1004707. doi: 10.1371/journal.ppat.1004707 (PMC4379056; doi:10.1371/journal.ppat.1004707)
Supplement: S3 Fig — (A) The ability of the various L. monocytogenes strains to invade, multiply, and spread from cell-to-cell was determined by assessing plaque formation within monolayers of fibroblast tissue culture cells. Cells were infected with an MOI of 10:1 and at 1 hour post-infection were washed, gentamicin was added, and plaques were visualized three days post-infection by staining with Neutral Red. Zones of clearing that did not stain indicate plaque formation. (B) Quantification of the diameter of plaques formed compared to wild-type size (set to 100%). At least 20 plaques from three independent experiments were counted and measured for each strain. Data shown for panels A and B are representative of three independent experiments done in duplicate. Loss of pplA but not the lipoprotein (pplA-G72stop mutant) impaired the ability of L. monocytogenes to reach the host cytosol and spread efficiently from cell-to-cell. (PDF) [file ppat.1004707.s003.pdf]

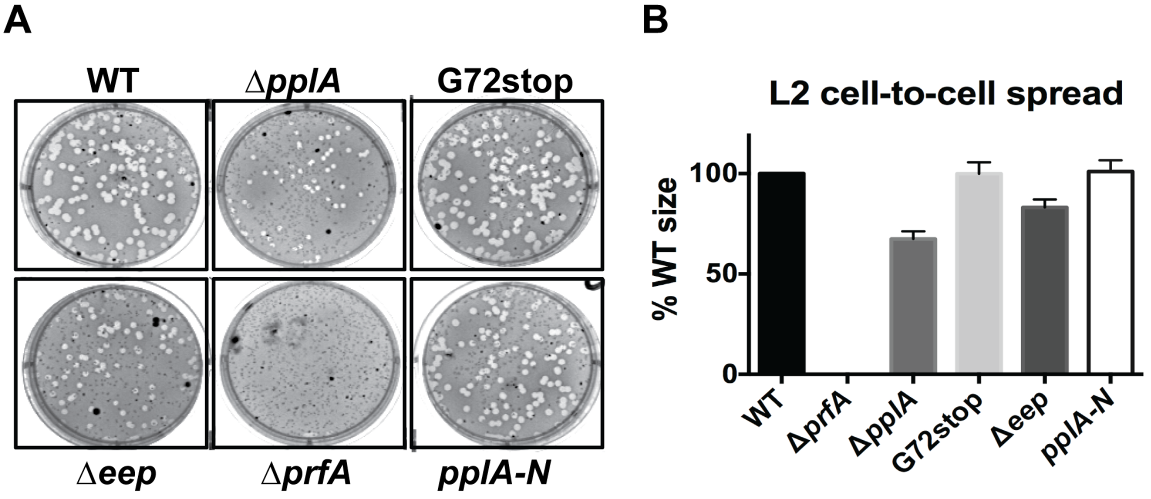

Supplemental Figure S3. **Loss of pPplA impairs the ability of *L. monocytogenes* to form plaques in cell monolayers.** **(A)** The ability of the various *L. monocytogenes* strains to invade, multiply, and spread from cell-to-cell was determined by assessing plaque formation within monolayers of fibroblast tissue culture cells. Cells were infected with an MOI of 10:1 and at 1 hour post-infection were washed, gentamicin was added, and plaques were visualized three days post-infection by staining with Neutral Red. Zones of clearing that did not stain indicate plaque formation. **(B)** Quantification of the diameter of plaques formed compared to wild-type (set to 100%). At least 20 plaques from three independent experiments were counted and measured for each strain. Data shown for panels A and B are representative of three independent experiments done in duplicate. Loss of *pplA* but not the lipoprotein (*pplA*-$G72_{STOP}$ mutant) impaired the ability of *L. monocytogenes* to reach the host cytosol and spread efficiently from cell-to-cell.
