# Supplementary material for: Identification of a Peptide-Pheromone that Enhances Listeria monocytogenes Escape from Host Cell Vacuoles
Source: PLoS Pathog. 2015 Mar 30;11(3):e1004707. doi: 10.1371/journal.ppat.1004707 (PMC4379056; doi:10.1371/journal.ppat.1004707)
Supplement: S4 Fig — Measurement of intracellular growth of wild-type and ΔpplA mutant in BMMØ using an MOI of 0.1:1. Macrophages were treated with 1 ng/mL IFNγ twenty-four hours prior to bacterial infection. Data shown is representative of three-independent experiments. (PDF) [file ppat.1004707.s004.pdf]

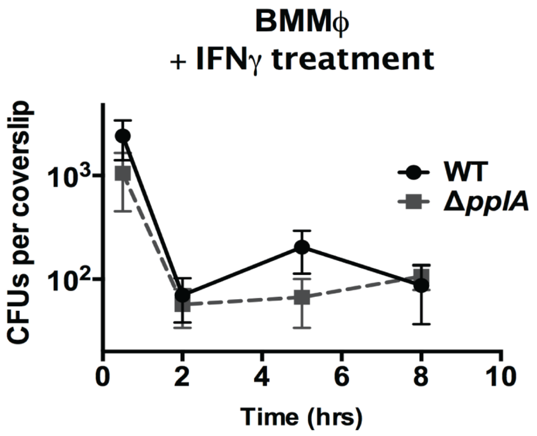

Supplemental Figure S4. **Δ*pplA* mutant and wild-type *L. monocytogenes* infection of IFNγ-treated bone marrow-derived macrophages (BMMØ).** Measurement of intracellular growth of wild type and *ΔpplA* in BMMØ using an MOI of 1:1. Macrophages were treated with 1 ng/mL IFNγ twenty-four hours prior to bacterial infection. Data shown is representative of three-independent experiments.
