# Supplementary material for: Identification of a Peptide-Pheromone that Enhances Listeria monocytogenes Escape from Host Cell Vacuoles
Source: PLoS Pathog. 2015 Mar 30;11(3):e1004707. doi: 10.1371/journal.ppat.1004707 (PMC4379056; doi:10.1371/journal.ppat.1004707)
Supplement: S5 Fig — (A) Two-dimensional gel electrophoresis of secreted proteins overnight to stationary phase cultures grown in BHI. Secreted proteins were TCA precipitated from bacterial culture supernatants and processed for 2-D gel analysis. B) Measurement of membrane integrity of bacterial strains grown in BHI with shaking overnight at 37°C to stationary phase. Bacterial cells were normalized to optical-density 600nm of 1.5, and cells were diluted 1:10 in PBS and stained with the LIVE/DEAD BacLight Viability Kit as per manufacture’s direction. Live bacterial cells with intact membranes fluoresce green due to the uptake of the membrane permeant SYTO9 dye, and dead cells or cells with compromised membranes incorporate the membrane impermeant propidium iodide (PI) dye and stain red. A minimum of 10 fields from two-independent experiments were visualized. (PDF) [file ppat.1004707.s005.pdf]

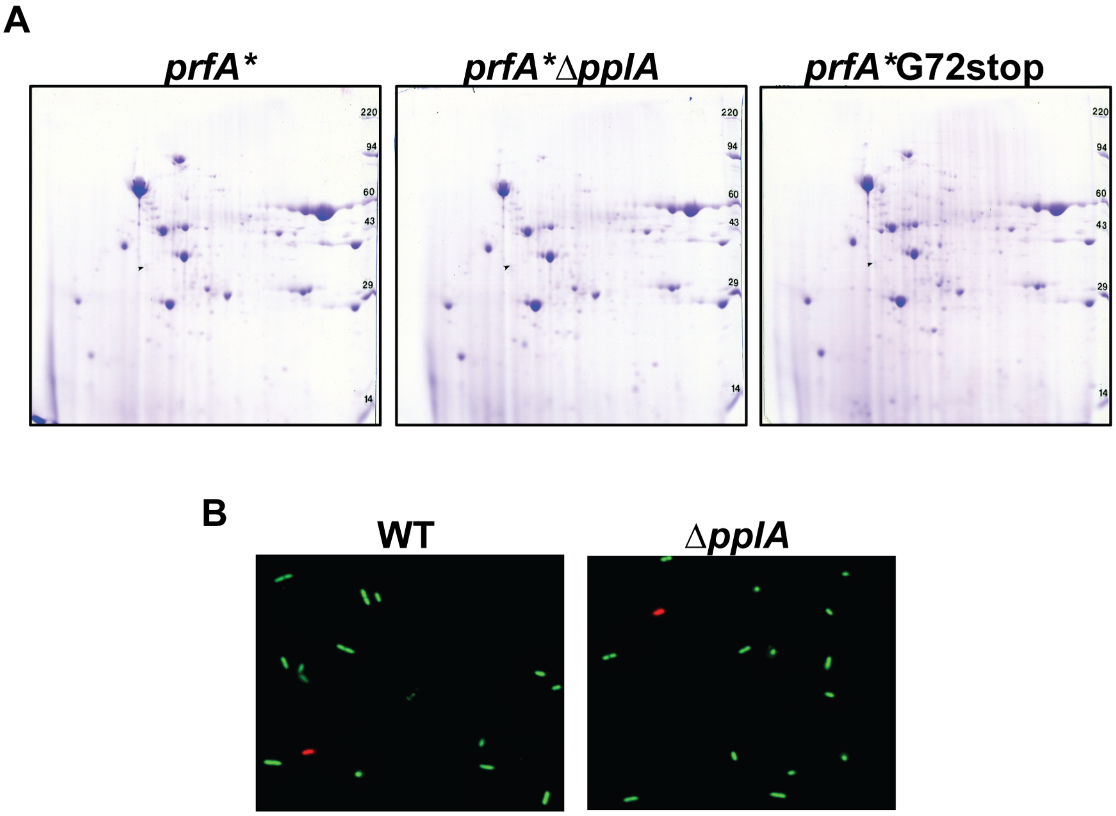

Supplemental Figure S5. ***L. monocytogenes prfA****, ***prfA****Δ***pplA*** **and** ***prfA* pplA*-G72$_{STOP}$ mutants display similar secreted protein profiles, and mutant bacteria do not exhibit increased cell lysis or decreased membrane integrity.** **(A)** Two dimensional gel electrophoresis of secreted protein preparations isolated from overnight stationary phase cultures grown in BHI. Secreted proteins were TCA precipitated from bacterial culture supernatants and processed for 2-D gel analysis. The *prfA**, *prfA* ΔpplA* and the *prfA* pplA*-G72$_{STOP}$ mutant protein profiles displayed similar patterns. **(B)** Measurement of membrane integrity of bacterial strains grown in BHI with shaking overnight at 37°C to stationary phase. Bacterial cells were normalized to optical density 600nm of 1.5, and cells were diluted 1:10 in PBS and stained with the LIVE/DEAD Bac*Light* Viability Kit as per manufacture's directions. Live bacterial cells with intact membranes fluoresce green due to the uptake of the membrane permeant SYTO9 dye, and dead cells or cells with compromised membranes incorporate the membrane impermeant propidium iodide (PI) dye and stain red. A minimum of 10 fields from two-independent experiments were visualized.
