# Supplementary material for: Identification of a Peptide-Pheromone that Enhances Listeria monocytogenes Escape from Host Cell Vacuoles
Source: PLoS Pathog. 2015 Mar 30;11(3):e1004707. doi: 10.1371/journal.ppat.1004707 (PMC4379056; doi:10.1371/journal.ppat.1004707)
Supplement: S6 Fig — Measurement of LLO-associated hemolytic activity as assessed by lysis of sheep red blood cells from serial dilutions of mixed culture supernatants of bacterial strains grown shaking in LB for 5 hours at 37°C. Hemolytic activity was determined as the reciprocal of supernatant dilution at which 50% lysis was observed and the data is reported as the percentage of WT, with WT values set to 100%. Assays were carried out using a 1:1 ratio of mixed culture supernatants to determine if supernatants derived from a Δhly mutant, which does not produce secreted LLO but still produces the pPplA peptide, could directly enhance lysis of RBC when added to supernatants derived from ΔpplA mutant, which does not contain peptide but contains LLO. Lysis activity was compared to ΔpplA culture supernatants mixed with supernatants from a Δhly ΔpplA double mutant (no secreted LLO or peptide). No enhancement of cell lysis was observed when supernatants containing peptide but no LLO were mixed with supernatants containing LLO but no peptide. Data is representative of three independent experiments. (PDF) [file ppat.1004707.s006.pdf]

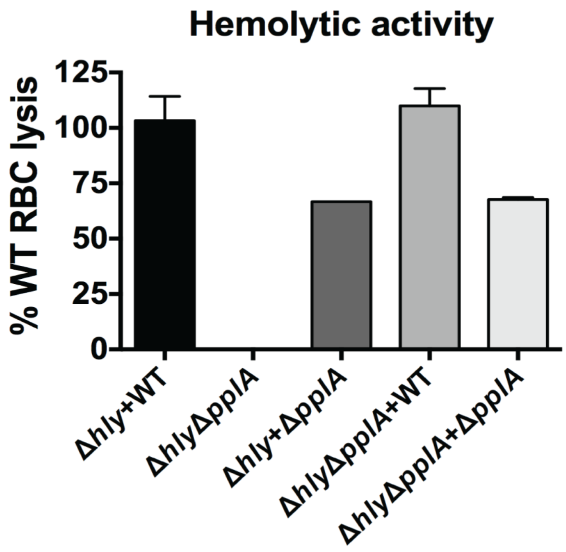

Supplemental Figure S6. pPlpA containing culture supernatants do not enhance LLO-dependent lysis of sheep red blood cells. Measurement of LLO-associated hemolytic activity as assessed by lysis of sheep red blood cells from serial dilutions of mixed culture supernatants of bacterial strains grown shaking in LB for 5 hours at 37°C. Hemolytic activity was determined as the reciprocal of supernatant dilution at which 50% lysis was observed and the data is reported as the percentage of WT, with WT values set to 100%. Assays were carried out using a 1:1 ratio of mixed culture supernatants to determine if supernatants derived from a Δ*hly* mutant, which does not produce secreted LLO but still produces the pPplA peptide, could directly enhance the lysis of RBC when added to supernatants derived from the Δ*pplA* mutant, which does not contain peptide but contains LLO. Lysis activity was compared to Δ*pplA* culture supernatants mixed with supernatants from a Δ*hly*Δ*pplA* double mutant (no secreted peptide). No enhancement of cell lysis was observed when supernatants containing peptide but no LLO were mixed with supernatants containing LLO but no peptide. Data is representative of three independent experiments.
