# Supplementary material for: Identification of a Peptide-Pheromone that Enhances Listeria monocytogenes Escape from Host Cell Vacuoles
Source: PLoS Pathog. 2015 Mar 30;11(3):e1004707. doi: 10.1371/journal.ppat.1004707 (PMC4379056; doi:10.1371/journal.ppat.1004707)
Supplement: S7 Fig — Assessment of bacterial aggregation in the presence of the antibiotic chloramphenicol, an inhibitor of protein translation. The optical-density at 600nm was monitored at the indicated points for wild-type, prfA*, or prfA* ΔpplA strains of stationary-phase bacteria resuspended in 1 mL of spent media derived from pplA-G72STOP cultures with or without the addition of 10 μg/mL of chloramphenicol during static incubation. Data shown is representative of at least two-independent experiments. (PDF) [file ppat.1004707.s007.pdf]

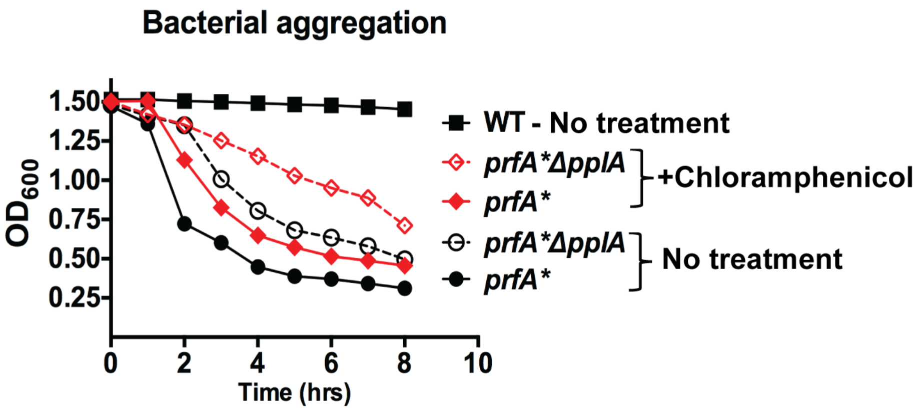

Supplemental Figure S7. Bacterial aggregation is reduced when protein synthesis is inhibited. Assessment of bacterial aggregation in the presence of the antibiotic chloramphenicol, an inhibitor of protein translation. The optical-density at 600nm was monitored at the indicated time points for wild type, *prfA**, or *prfA* ΔpplA* strains of log-phase bacteria resuspended in 1 mL of spent media derived from *prfA** strains, with or without the addition of 10 µg/mL of chloramphenicol during static incubation. Data shown is representative of at least two-independent experiments.
