# Supplementary material for: Identification of a Peptide-Pheromone that Enhances Listeria monocytogenes Escape from Host Cell Vacuoles
Source: PLoS Pathog. 2015 Mar 30;11(3):e1004707. doi: 10.1371/journal.ppat.1004707 (PMC4379056; doi:10.1371/journal.ppat.1004707)
Supplement: S8 Fig — Swimming motility was assessed on semisolid (0.3% w/v agar) BHI media. Plates were inoculated with 5 μL of the indicated L. monocytogenes strains grown overnight to stationary phase in BHI with shaking and normalized to an optical-density 600nm of 1.5. The inoculated plates were then incubated overnight at 37°C. Swimming motility is evident from the migration of the bacteria away from the original spot of inoculation. Data shown is representative of at least three-independent experiments. (PDF) [file ppat.1004707.s008.pdf]

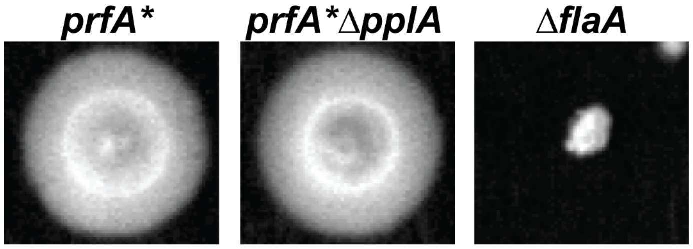

Supplemental Figure S8. Loss of pPplA does not impair flagellar swimming motility in a *prfA background strain**. Swimming motility was assessed on semisolid (0.3% w/v agar) BHI media. Plates were inoculated with 5 µL of the indicated *L. monocytogenes* strains grown overnight to stationary phase in BHI with shaking and normalized to an optical-density at 600nm of 1.5. The inoculated plates were then incubated overnight at 37°C. Swimming motility is evident from the migration of the bacteria away from the original spot of inoculation. Data shown is representative of at least three-independent experiments.
